# Supplementary material for: Structural Basis of Phosphatidic Acid Sensing by APH in Apicomplexan Parasites
Source: Structure. 2018 Aug 7;26(8):1059–1071.e6. doi: 10.1016/j.str.2018.05.001 (PMC6084407; doi:10.1016/j.str.2018.05.001)
Supplement: Document S1. Figures S1–S6 and Tables S1–S3 [file mmc1.pdf]

Structure, Volume *26*

Supplemental Information

Structural Basis of Phosphatidic Acid Sensing by APH in Apicomplexan Parasites

Nick Darvill, David J. Dubois, Sarah L. Rouse, Pierre-Mehdi Hammoudi, Tom Blake, Stefi Benjamin, Bing Liu, Dominique Soldati-Favre, and Steve Matthews

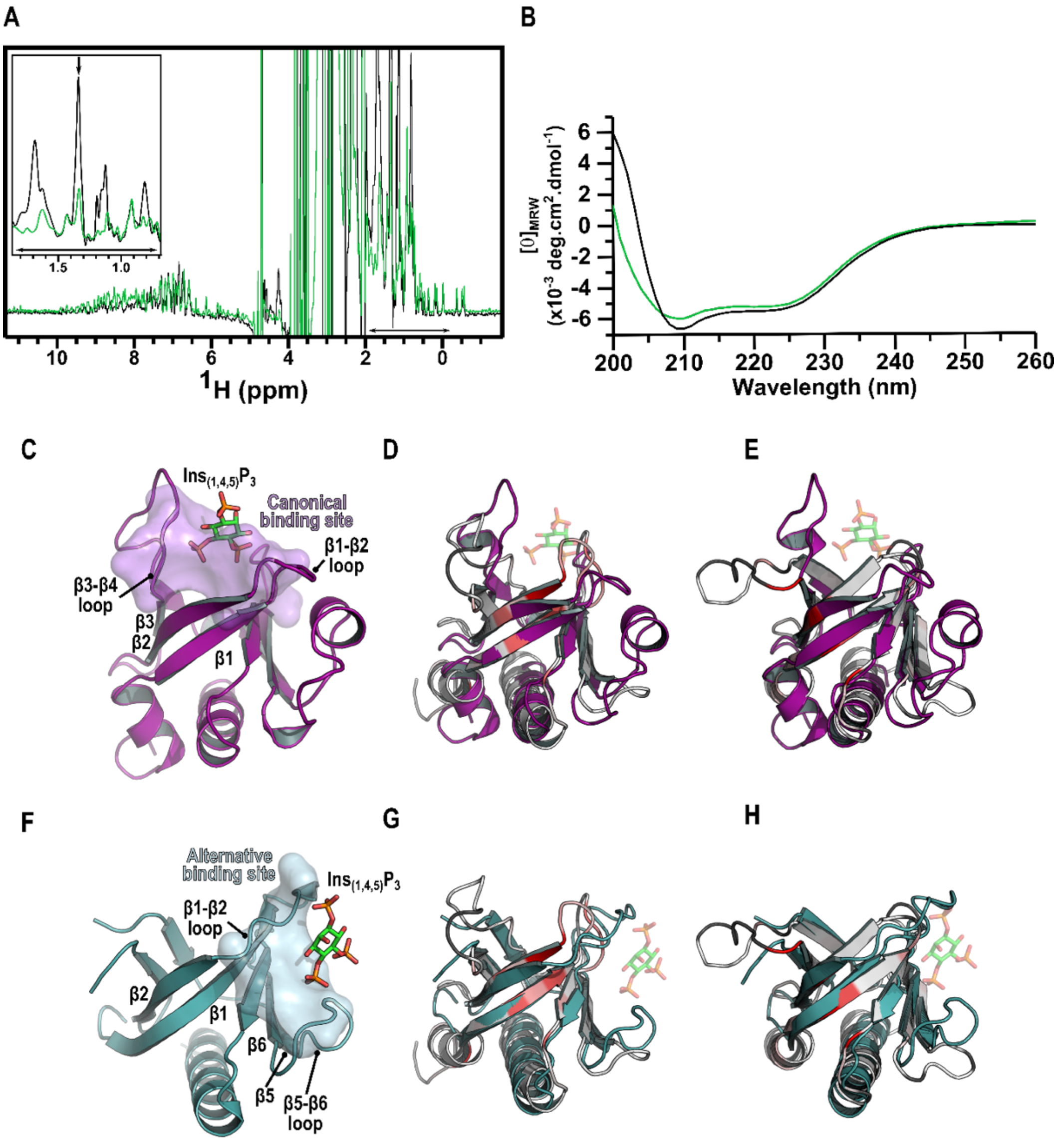

SI Figure S1 **(Related to Figure 1 and 2). Comparison between TgAPH$_{22\text{-}229}$ and TgAPH$_{99\text{-}229}$ HSQC spectra, and APH compared to canonical and alternative binding PH domains.** A) Overlay of 1D NMR spectra for TgAPH99-229 (green) and TgAPH22-229 (black). Inset, enlarged 1D spectral region highlights the peak corresponding to Ala methyl groups (indicated by a black arrow) that in TgAPH22-229, has a lower intensity than expected. B) CD spectra for TgAPH99-229 (green) and TgAPH22-229 (black). C) Cartoon representation of the crystal structure of the PH domain from R.norvegicus PLC_δ1 (PDB ID 1MAI) bound to inositol 1,4,5-triphosphate (Ins($_{1,4,5}$)P$_3$) as an example of a canonical binding PH domain. The canonical binding site is represented as a purple semi-transparent surface. D) and E) PLC_δ1 PH domain aligned to PfAPH$_{106\text{-}235}$ and TgAPH$_{99\text{-}229}$ respectively with bound Ins($_{1,4,5}$)P$_3$ shown for reference to the canonical binding site. F) Cartoon representation of the crystal structure of the PH domain from human ArhGAP9 (PDB ID 2p0D) bound to Ins($_{1,4,5}$)P$_3$ as an example of an alternative binding PH domain. The alternative binding site is represented as a cyan semi-transparent surface. G) and H) ArhGAP9 PH domain aligned to PfAPH$_{106\text{-}235}$ and TgAPH$_{99\text{-}229}$ respectively with bound Ins($_{1,4,5}$)P$_3$ shown for reference to the alternative binding site.

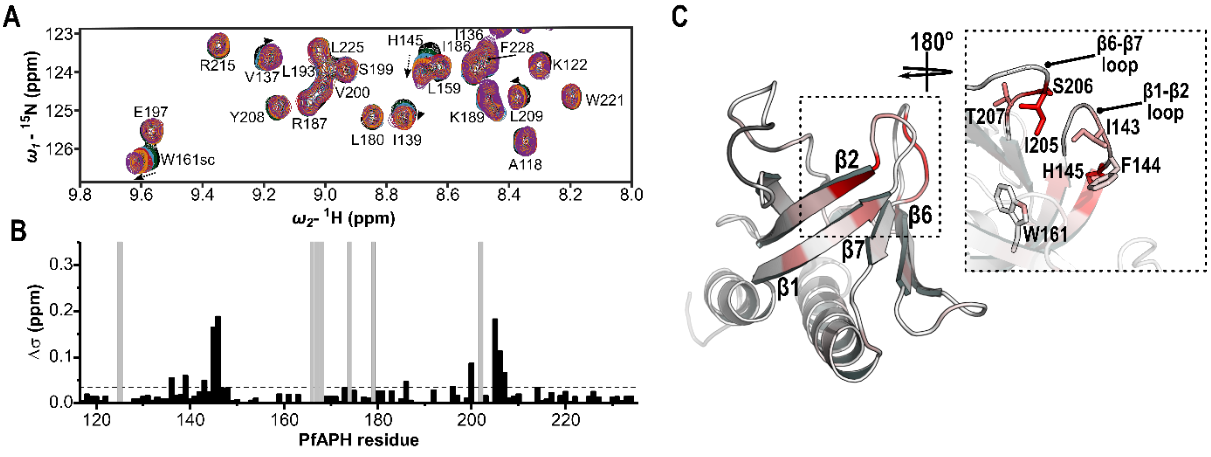

SI Figure S2 **(Related to Figure 2). $PfAPH_{106-235}$ titration with PA enriched bicelles.** A) Representative data showing overlay of ^{15}N-labelled $PfAPH_{106-235}$ 2D ^{1}H-^{15}N HSQC spectra in the presence of increasingly PA enriched bicelles with the following DHPC:DMPC:POPA composition: *black*, 75%:25%:25%; *green*, 75%:24%:1%; *blue*, 75%:18.75%:6.25%; *red*, 75%:16.25%:8.75%; *orange*, 75%:12.5%:12.5%; *purple*, 75%:10%:15%. B) Plot of CSPs observed in A) upon titration with DHPC:POPC:POPA (75%:12.5%:12.5%) bicelles, versus $PfAPH_{106-235}$ sequence number, residues that could not be assigned are indicated by a grey bar. Prominent CSP's are categorised as greater than 2σ's from the mean noise (0.035ppm), which is represented by a dotted line. C) CSPs mapped onto the structure of $PfAPH_{106-235}$, coloured in a 20 interval *red* spectrum. A more intense colouring indicates a greater CSP as each interval represents 0.5σ from the mean noise. Key residues clustered around the β1-β2 and β6-β7 loops are labelled, unassigned residues are coloured *dark grey*.

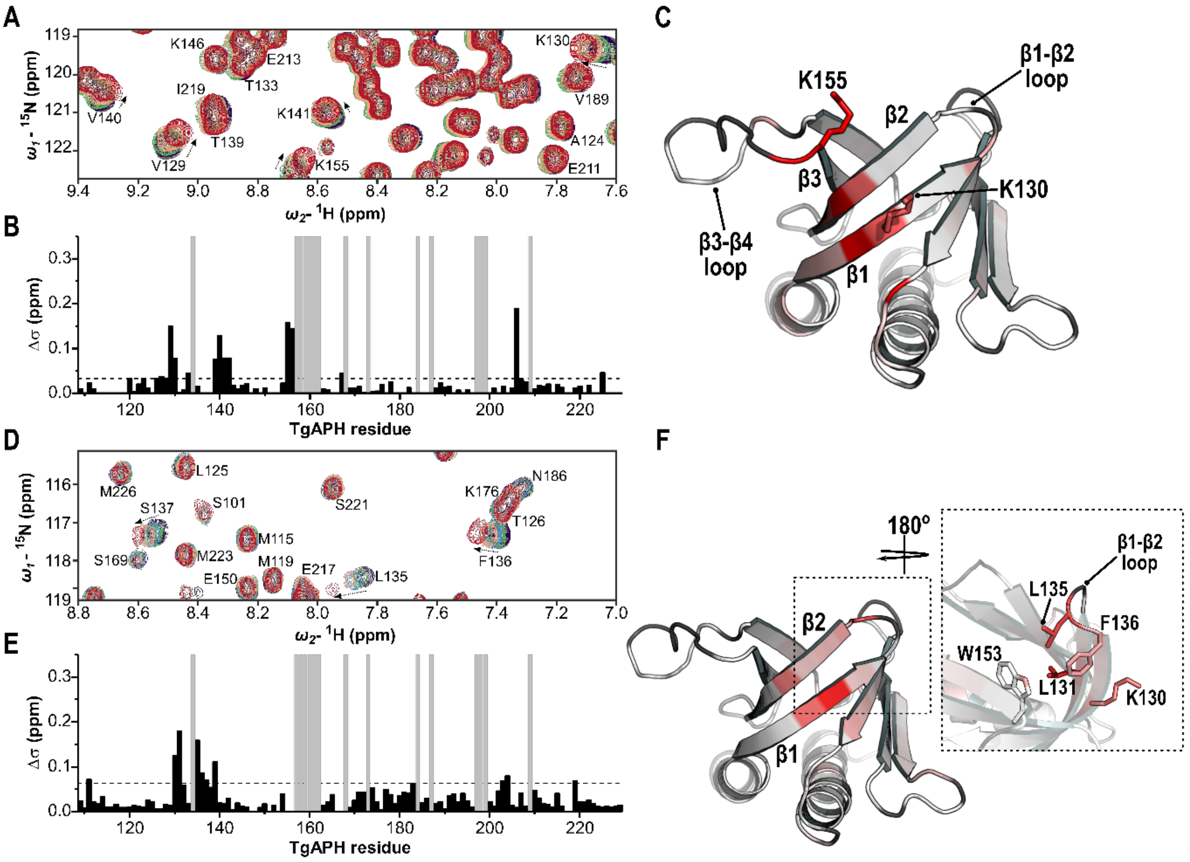

SI Figure S3 **(Related to Figure 2). Mapping the TgAPH$_{99-229}$:PA interface.** A) Overlay of representative ^{15}N-labelled TgAPH$_{99-229}$ 2D ^{1}H-^{15}N HSQC spectra recorded upon titration with increasing molar ratios of short-chain PA. HSQC spectra are coloured according to the molar ratio between ^{15}N-labelled TgAPH$_{99-229}$ and short-chain PA; *grey* 1:0, *purple* 1:1, *green* 1:3, *orange* 1:7, *red* 1:15. B) Plot of CSPs observed in A) upon titration with 15-fold molar excess of short-chain PA, versus TgAPH$_{99-229}$ sequence number. Residues that could not be assigned are indicated by a grey bar. Prominent CSP's are categorised as greater than 2σ's from the mean noise (0.032ppm), which is represented by a dotted line. C) CSPs observed in A) mapped onto the structure of TgAPH$_{99-229}$, coloured in a 20 interval *red* spectrum. A more intense colouring indicates a greater CSP as each interval represents 0.5σ from the mean noise. Key residues clustered around the β1 strand and β3-β4 loop region are labelled, unassigned residues are coloured *dark grey*. D) Representative data showing overlay of ^{15}N-labelled TgAPH$_{99-229}$ 2D ^{1}H-^{15}N HSQC spectra in the presence of increasingly PA enriched bicelles with the following DHPC:DMPC:POPA composition: *purple*, 75%:12.5%:12.5%; *green*, 75%:24%:1%; *blue*, 75%:18.75%:6.25%; *orange*, 75%:16.25%:8.75%; *red*, 75%:12.5%:12.5%. E) Plot of CSPs observed in D) upon titration with DHPC:POPC:POPA (75%:12.5%:12.5%) bicelles, versus PfAPH$_{106-235}$ sequence number, residues that could not be assigned are indicated by a grey bar. Prominent CSP's are categorised as greater than 2σ's from the mean noise (0.062ppm), which is represented by a dotted line. F) CSPs observed in D) mapped onto the structure of TgAPH$_{99-229}$, coloured as in C). Key residues clustered around the β1-β2 loop are labelled.

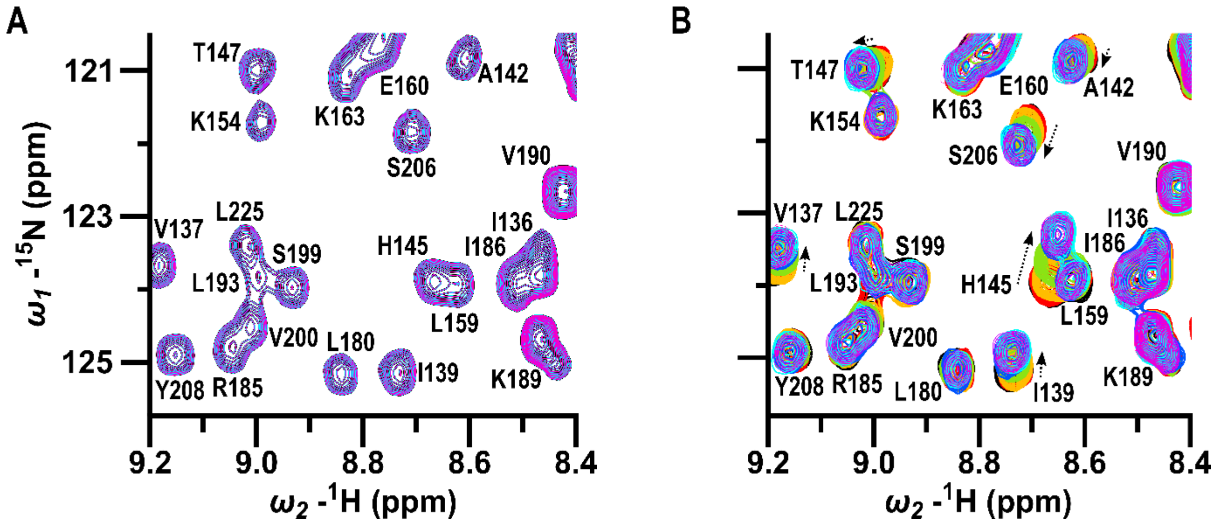

SI Figure S4 **(Related to Figure 2). APH does not bind PI(4,5)P2 and is incapable of dual lipid recognition.** A) Overlay of representative ^{15}N- PfAPH$_{106-235}$ 2D ^{1}H-^{15}N HSQC spectra recorded upon titration with increasing molar ratios of phosphatidylinositol 4,5 bisphosphate (PI(4,5)P2). HSQC spectra are coloured according to the molar ratio between ^{15}N-labelled PfAPH and PI(4,5)P2; *black* 1:0, *cyan* 1:1, *purple* 1:2, *magenta* 1:4. HSQC spectra have decreasing line thickness to illustrate that peaks are overlaid. B) Overlay of representative ^{15}N-labelled PfAPH$_{106-235}$ 2D ^{1}H-^{15}N HSQC spectra recorded upon initial titration with increasing molar ratios of short-chain PA and then titration with increasing molar ratios of PI(4,5)P2. HSQC spectra are coloured according to the molar ratio between ^{15}N-labelled PfAPH$_{106-235}$, short-chain PA and PI(4,5)P2; *black* 1:0:0, *red* 1:1:0, *orange* 1:3:0, *lime* 1:7:0, *blue* 1:15:0, *cyan* 1:15:1, *purple* 1:15:2, *magenta* 1:15:8. Note that CSP's are only observed for peaks upon titration with short-chain PA.

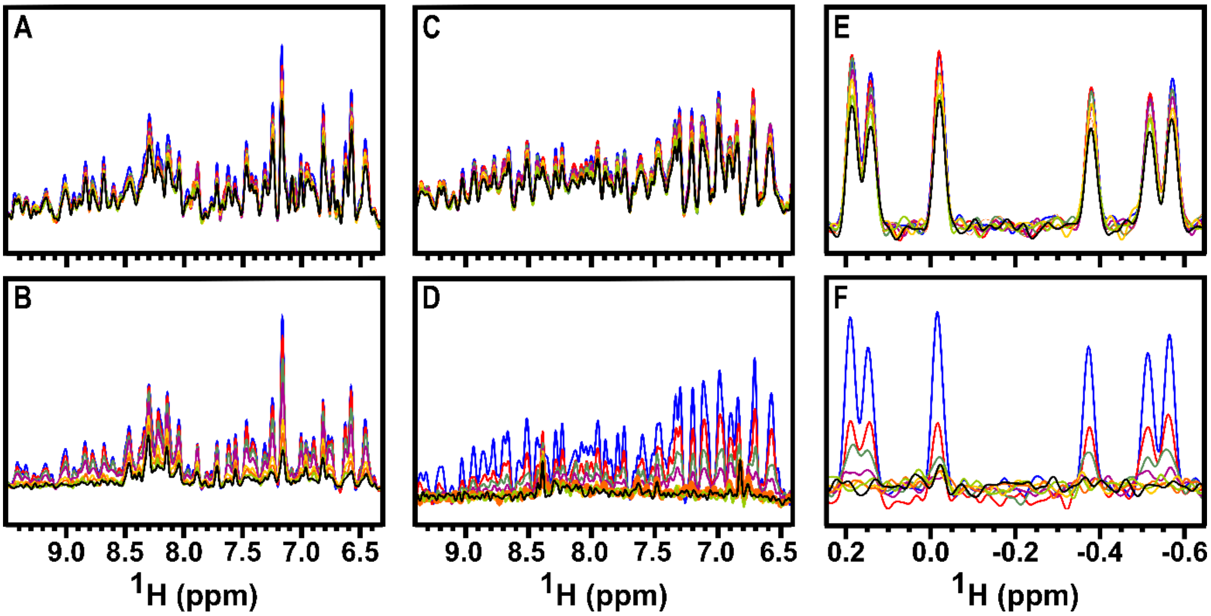

SI Figure S5 **(Related to Figure 3). APH titration with SUVs.** A) PfAPH$_{106\text{-}235}$ 1D ^{1}H NMR spectral region corresponding to the downfield-shifted amide region (9.5 to 6.3ppm) recorded upon titration with increasing concentration of SUVs composed of A) POPC (100%) or B) POPC and POPA (50%:50%). TgAPH$_{99\text{-}229}$ 1D ^{1}H NMR spectral region corresponding to the downfield-shifted amide region (9.4 to 6.4ppm) recorded upon titration with increasing concentration of SUVs composed of C) POPC (100%) or D) POPC and POPA (50%:50%). E) and F) identical to C) and D) but for TgAPH$_{99\text{-}229}$ 1D ^{1}H NMR spectral region corresponding to the upfield-shifted methyl region (0.25 to -0.65ppm). APH:SUVs (total lipid) molar ratio: *blue*, free APH in solution; *red* 1:2; *green* 1:4; *purple* 1:7; *yellow* 1:15; *orange* 1:20; *lime* 1:25; *black* 1:30.

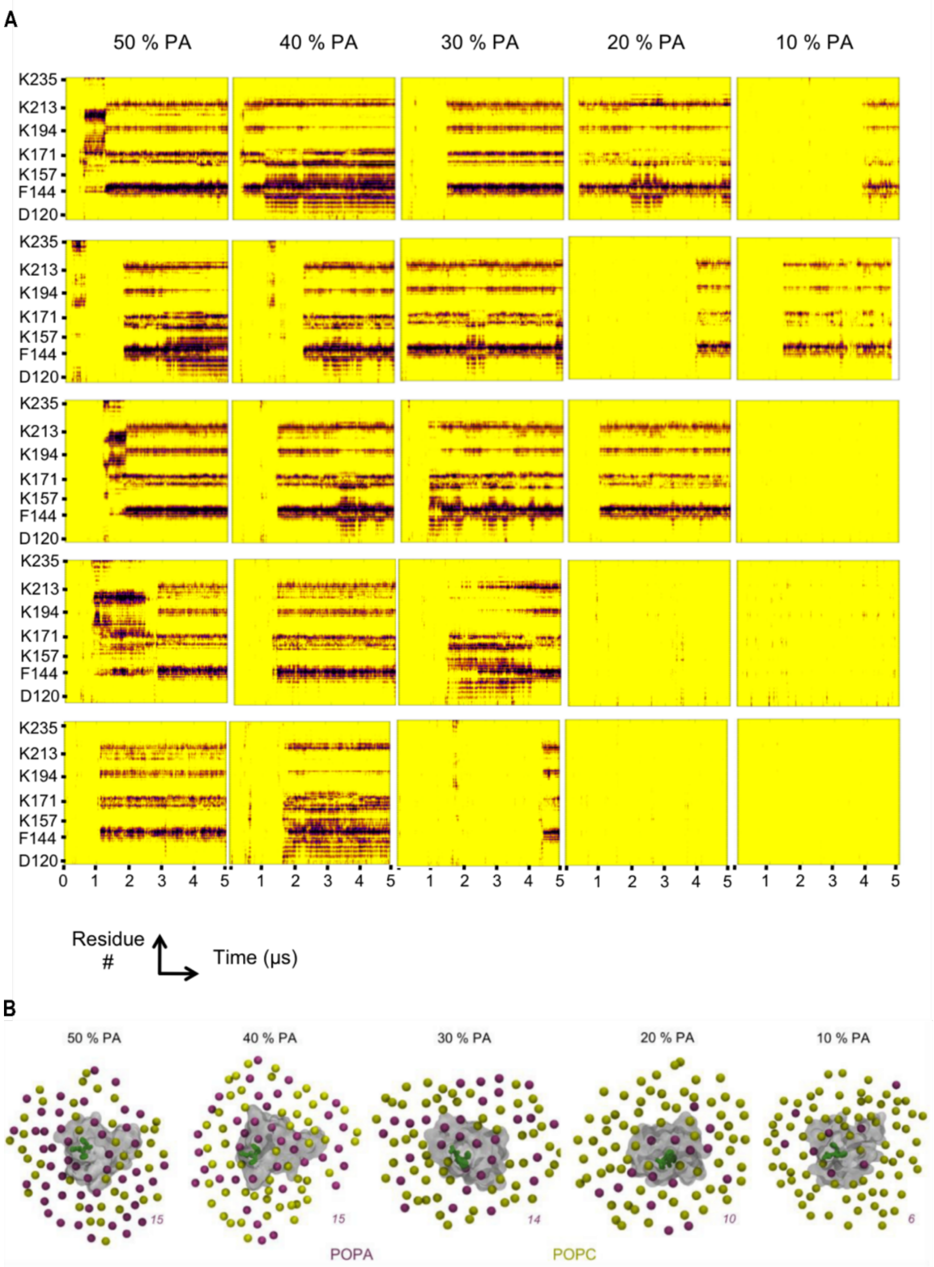

SI Figure S6 **(Related to Figure 6). Close contact plots between PfAPH$_{106\text{-}235}$ residues and POPA lipid headgroups, and clustering of POPA around the PfAPH$_{106\text{-}235}$ surface.** A) Residues are coloured purple when the distance from a POPA headgroup particle is below a cut-off of 1 nm, shown for 5 different simulations. The pattern of contacts between the protein and POPA is consistently observed at all 5 concentrations of POPA, indicating that PfAPH$_{106\text{-}235}$ predominantly adopts a single, stable orientation within the membrane. Upon finding the most favourable bound orientation the protein is not observed to depart the membrane for the remainder of the simulation time. B) Representative snapshots at each membrane composition (50- 10% PA) of the bound protein and lipid headgroup phosphate particles are shown. POPA headgroups are shown as purple spheres and POPC in yellow. The protein is shown as a grey surface with the buried hydrophobic residues I143-H145 shown as green ball and sticks. The POPA headgroups (purple) are observed to cluster together in the region of PfAPH$_{106\text{-}235}$ at all POPA concentrations. The maximum number of POPA within 1 nm of the protein surface for each PA composition is shown.

SI Table S1 **(Related to STAR Methods). Plasmids used in this study.**

Plasmid	Description	Source
For expression of full length APH and C-terminal PH domain		
pNIC28a-Bsa4_*PfAPH*$_{106\text{-}235}$	*6xHis-TEV-PfAPH*$_{106\text{-}235}$	This paper
pNIC28a-Bsa4_*TgAPH*$_{99\text{-}229}$	*6xHis-TEV-TgAPH*$_{99\text{-}229}$	This paper
pNIC28a-Bsa4_*TgAPH*$_{22\text{-}229}$	*6xHis-TEV-TgAPH*$_{22\text{-}229}$	This paper
For functional characterization of TgAPH in T. gondii		
iKD-GAC-DHFR	iKD-template	(Jacot et al., 2016)
pSAG1::CAS9-GFP-U6::sgUPRT	UPRT-Cas9 guide	(Shen et al., 2014)
pSAG1::CAS9-GFP-U6::sg*TgAPH*	*TgAPH*-Cas9 guide	This paper
pT8-N21-Ty-APH-BleO	Intermediate plasmid	(Bullen et al., 2016)
5'UPRT-pT8-MycGFPPfMyoAtail-Ty-3'UPRT	Intermediate plasmid	(Jacot et al., 2016)
pTub5-CAT	Intermediate plasmid	(Kim et al., 1993)
5'UPRT-CAT-pT8-N21-Ty-APH-3'UPRT	*TgAPH*-N21Ty	This paper
5'UPRT-CAT-pT8-N21-Ty-APH-K130A+K132A-3'UPRT	*TgAPH*-N21Ty-K130A+K132A	This paper
5'UPRT-CAT-pT8-N21-Ty-APH-L135A+F136A-3'UPRT	*TgAPH*-N21Ty-L135A+F136A	This paper
5'UPRT-CAT-pT8-N21-Ty-APH-K155A+K157A-3'UPRT	*TgAPH*-N21Ty-L155A+F157A	This paper
5'UPRT-CAT-pT8-N21-Ty-APH-E138A-3'UPRT	*TgAPH*-N21Ty-L138A	This paper
5'UPRT-CAT-pT8-N21-Ty-APH-Δ-linker-3'UPRT	*TgAPH*-N21Ty-Δ-linker	This paper
5'UPRT-CAT-pT8-N21-Ty-APH-Sc-linker-3'UPRT	*TgAPH*-N21Ty-Sc-linker	This paper

SI Table S2 **(Related to STAR Methods). Oligonucleotides used in this study.**

Oligonucleotide	Sequence (5'→3')
For expression of full length APH and C-terminal PH domain	
Fw_PfAPH	TACTTCCAATCCATGAAACTGAGCACCGAT
Rv_PfAPH	TATCCACCTTTACTGTCATTTCATGCTCATAATTTTGC
Fw_TgAPH	TACTTCCAATCCATGTCTGAACCTGACAACG

Rv_TgAPH	TATCCACCTTTACTGTTATTTCATCGACATGAACT
Fw_TgAPH_fl	TACTTCCAATCCATGCACATCAAAGCGAAAACC
Rv_TgAPH_fl	TATCCACCTTTACTGTCATTTCATCGACATGAACTTCAT

Site directed mutagenesis of APH PH domain

PfAPH_K138A_K140A_Fw	TGCAACCGCGATTTTTCATGAAAC
PfAPH_K138A_K140A_Rv	ATTGCCACAATTTTGGTCAGGGTTTTG
PfAPH_K163A_K165A_Fw	CGCAAACGATAGCGATGGCAAA
PfAPH_K163A_K165A_Rv	CCTGCATACCATTCCAGCATTTTG
PfAPH_I143A_F144A_Fw	TAAAACCGCGGCTGCTCATGAAACCGTG
PfAPH_I143A_F144A_Rv	ATTTTCACAATTTTGGTCAG
PfAPH_H145A_Fw	CGCGATTTTTGCTGAAACCGTGAAAG
PfAPH_H145A_Rv	GTTTTAATTTTCACAATTTTGGTC
PfAPH_E146A_Fw	GATTTTTCATGCAACCGTGAAAG
PfAPH_E146A_Rv	GCGGTTTTAATTTTCACAATTTTG
TgAPH_K130A_K132A_Fw	CGCGACACACCTCTTTTCTGAG
PfAPH_K130A_K132A_Rv	AGCGCGACAACTTTAGTAAGAGCG
TgAPH_K155A_K157A_Fw	CAAGACACACGCCGCTTCTGAGACAGTGAAG
TgAPH_K155A_K157A_Rv	AGCTTGACAACTTTAGTAAG
TgAPH_L135A_F136A_Fw	CAAGACACACGCCGCTTCTGAGACAGTGAAG
TgAPH_L135A_F136A_Rv	AGCTTGACAACTTTAGTAAG
TgAPH_E138A_Fw	CTCTTTTCTGCGACAGTGAAGG
TgAPH_E138A_Rv	GTGTGTCTTGAGCTTGAC

Functional characterization of TgAPH in T. gondii

CAT-Rev_2170	GCCCCGCCCTGCCACTCATCGC
M13-Rev_4749	AACAGCTATGACCATG
gRNA_4883	AACTTGACATCCCCATTTAC
TgAPH_6324 (iKD)	TCCCCACTGCGCATTATTTTGCTTCCACTTCATGTTTGCGGATCCGGGG

TgAPH_6325 (iKD)	ACCTGTCAAAGCAGCTCATAGTGTTCCCCATTTTGATATCCCTAGGAATTCAC
TgAPH_6326 (APH CRISPR guide)	GCGTCGCTGAACCCGCAGTAGTTTTAGAGCTAGAAATAGC
TgAPH_6339Fw (K130A+K132A)	CGCGACACACCTCTTTTCTGAG
TgAPH_6529 Rv (K130A+K132A)	AGCGCGACAACTTTAGTAAGAGCG
TgAPH_6341 Fw (L135A+F136A)	CAAGACACACGCGGCGTCTGAGACAGTGAAGG
TgAPH_6342 Rv (L135A+F136A)	AGCTTGACAACTTTAGTAAG
TgAPH_6343 Fw (K155A+K157A)	TGCGAGTACCGCCGGCGCCCAGGAC
TgAPH_6344 Rv (K155A+K157A)	CCCGCGAACCACTGAACCTCTTCTCCATCTTTGCTG
TgAPH_6423 Fw (linker deletion)	TCTGAACCTGACAACGATGCGG
TgAPH_6424 Rv (linker deletion)	GGGAGCAGCGGCTCCAGG
UPRT_6611	CTCAAGTCTCAAAAGCAGATCCGC
UPRT_6610	ATCCCCTTCATTTTGCTTACGCAG
TgAPH _7327 Rv (E138A)	TCTTTTCTGCGACAGTGAAGG
TgAPH _7368 Rv (E138A)	AGAAAAGAGGTGTGTCTTGAGCTT
TgAPH _7399 Fw (Sc-linker)	CCCCCCGGGGATCTGCGCAGCAGCCAGATGGAAGCGAAAATGCGCTATGATGAAAAATCTGAACCTGACAACGATGCGG
TgAPH_7400 Rv (Sc-linker)	CCCCCCGGGTTCGCGCACGCTGCTGGTCGGGCGCATGCTTTCCAGGGTTTTGCTGCTGGGAGCAGCGGCTCCAGG

SI Table S3 **(Related to STAR Methods). 1D ^{1}H-NMR LUV titration experiments.**

**Volume of 8mM LUV stock added to NMR sample (µl)**	**LUV concentration (µM)**	**APH:LUV ratio**
0.0	0	1:0
6.9	100	1:2
7.0	200	1:4
10.6	350	1:7
28.7	750	1:15
18.8	1000	1:20
19.4	1250	1:25
20.0	1500	1:30
