# Supplementary material for: Structural Basis of Phosphatidic Acid Sensing by APH in Apicomplexan Parasites
Source: Structure. 2018 Aug 7;26(8):1059–1071.e6. doi: 10.1016/j.str.2018.05.001 (PMC6084407; doi:10.1016/j.str.2018.05.001)
Supplement: Document S2. Article plus Supplemental Information [file mmc2.pdf]

Article

Structure

Structural Basis of Phosphatidic Acid Sensing by APH in Apicomplexan Parasites

Graphical Abstract

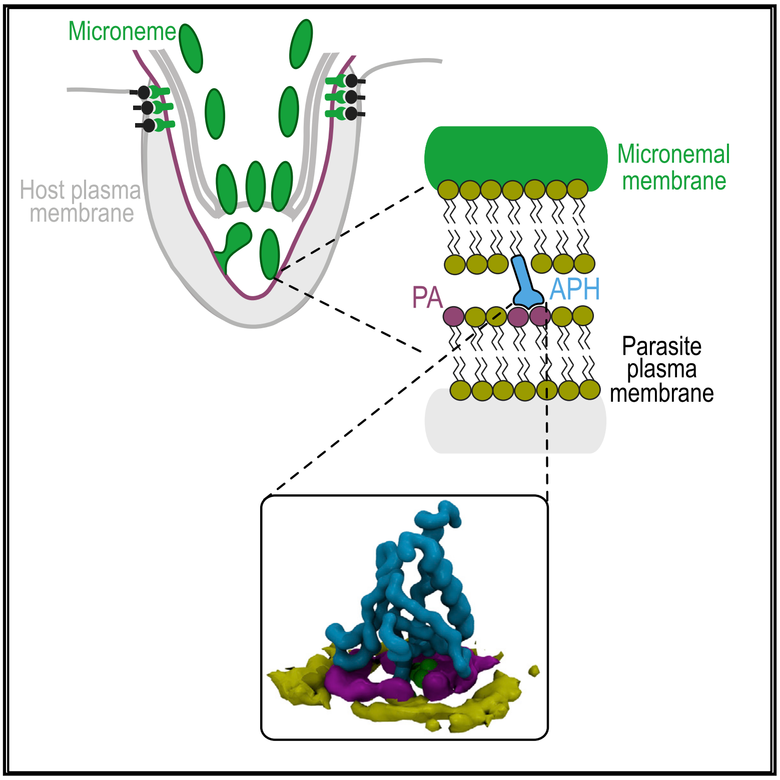

Authors

Nick Darvill, David J. Dubois, Sarah L. Rouse, ..., Bing Liu, Dominique Soldati-Favre, Steve Matthews

Correspondence

dominique.soldati-favre@unige.ch (D.S.-F.), s.j.matthews@imperial.ac.uk (S.M.)

In Brief

High-resolution structures of a phosphatidic acid effector protein (APH) from both *Plasmodium falciparum* and *Toxoplasma gondii* parasites and its interaction with the lipid mediator unravel a new class of PH domain that initiates microneme-plasma membrane fusion by inserting and clustering phosphatidic acid within membranes.

Highlights

- Solution structures of APH from *Toxoplasma gondii* and *Plasmodium falciparum*
- APH represents a new class of PH domain
- APH phosphatidic acid binding site encompasses canonical and atypical sites
- APH inserts into the bilayer and clusters multiple phosphate head-groups

Darvill et al., 2018, Structure *26*, 1059–1071
August 7, 2018 © 2018 The Authors. Published by Elsevier Ltd.
https://doi.org/10.1016/j.str.2018.05.001

CellPress

Structural Basis of Phosphatidic Acid Sensing by APH in Apicomplexan Parasites

Nick Darvill,[1,4] David J. Dubois,[2,4] Sarah L. Rouse,[1] Pierre-Mehdi Hammoudi,[2] Tom Blake,[1] Stefi Benjamin,[1] Bing Liu,[1,3] Dominique Soldati-Favre,[2,*] and Steve Matthews[1,3,5,*]
[1]Department of Life Sciences, Faculty of Natural Sciences, Imperial College London, London SW7 2AZ, UK
[2]Department of Microbiology & Molecular Medicine, Faculty of Medicine, University of Geneva, 1 Rue Michel-Servet, 1211 Geneva, Switzerland
[3]BioBank, First Affiliated Hospital, School of Medicine, Xi'an Jiaotong University, Xi'an, 710049, P. R. China
[4]These authors contributed equally
[5]Lead Contact
*Correspondence: dominique.soldati-favre@unige.ch (D.S.-F.), s.j.matthews@imperial.ac.uk (S.M.)
https://doi.org/10.1016/j.str.2018.05.001

SUMMARY

Plasmodium falciparum **and** ***Toxoplasma gondii*** **are obligate intracellular parasites that belong to the phylum of Apicomplexa and cause major human diseases. Their access to an intracellular lifestyle is reliant on the coordinated release of proteins from the specialized apical organelles called micronemes and rhoptries. A specific phosphatidic acid effector, the acylated pleckstrin homology domain-containing protein (APH) plays a central role in microneme exocytosis and thus is essential for motility, cell entry, and egress. TgAPH is acylated on the surface of the micronemes and recruited to phosphatidic acid (PA)-enriched membranes. Here, we dissect the atomic details of APH PA-sensing hub and its functional interaction with phospholipid membranes. We unravel the key determinant of PA recognition for the first time and show that APH inserts into and clusters multiple phosphate head-groups at the bilayer binding surface.**

INTRODUCTION

Apicomplexans form a group of parasitic protists that includes agents of major human diseases: *Toxoplasma gondii* responsible for toxoplasmosis (Robert-Gangneux and Darde, 2012) and *Plasmodium* species causing malaria (Bhatt et al., 2015). Among the five species of *Plasmodium* capable of infecting humans, *Plasmodium falciparum* is responsible for the most severe form of malaria, particularly in endemic areas of sub-Saharan Africa where ~90% of global malaria-related deaths occur (Kim and Schneider, 2013). The intracellular lifestyle of apicomplexan parasites (Cowman and Crabb, 2006) is reliant on the actions of proteins released from specialized apical organelles, known as micronemes and rhoptries (Santos and Soldati-Favre, 2011). These apical secretory organelles critically contribute to gliding motility, invasion, and egress from infected cells. Notably, the micronemes secrete a perforin to egress from infected cells (Roiko and Carruthers, 2013). Several adhesins are also secreted to promote parasite attachment to the target cell and the formation of a moving junction between the cell and the actomyosin system, which drives the parasite inside the host-cell vacuole.

The signaling pathway leading to microneme secretion is complex and involves changes in potassium and cyclic nucleotide concentration levels that lead to an increase in parasite intracellular calcium levels (Brochet et al., 2014; Carruthers and Sibley, 1999; Moudy et al., 2001). Phosphoinositide-phospholipase C (PI-PLC) plays a central role in the signaling cascade leading to microneme secretion (Singh and Chitnis, 2012). PI-PLC catalyzes the conversion of $PI_{(4,5)}P_2$ into IP_3 and diacylglycerol (DAG), which is further converted into phosphatidic acid (PA) via the activity of the specific diacylglycerol kinase 1 (DGK1) at the parasite plasma membrane, while IP_3 is thought to stimulate a rise in cytosolic Ca^{2+} concentration (Bullen et al., 2016).

The discovery that changes in PA levels play an important role in controlling microneme exocytosis uncovered the identification of a novel PA sensor, conserved across the Apicomplexa and that possesses N-terminal lipid anchors and a predicted phospholipid binding domain (Bullen et al., 2016). This protein named acylated pleckstrin homology (PH) domain-containing protein (APH), is anchored at the surface of the micronemes via N-terminal myristoylation and palmitoylation. *T. gondii* and *P. falciparum* APH (TgAPH and PfAPH) bind selectively to PA both on PIP-strips and in liposome assays. It was proposed that this bipartite interaction tethers the microneme and plasma membranes together and participates in organelle fusion (Bullen et al., 2016) via the involvement of SNARE-like proteins (Figures 1A–1C), such as DOC2.1 (Farrell et al., 2012; Jean et al., 2014). The broader importance of PA in motility, invasion, and egress has been further highlighted by recent studies. The first is the discovery of the glideosome-associated connector protein (GAC), which links key microneme protein complexes to the actomyosin system and involves a specific interaction with PA via the C-terminal PH domain of GAC (Jacot et al., 2016). In a second example, new structural insight has been provided for the conserved protein CelTOS, which is a promising vaccine candidate (Pirahmadi et al., 2018). CelTOS is essential for parasite traversal of cells, and has been shown to bind to and disrupt PA-rich membranes (Jimah et al., 2016).

© 2018 The Authors. Published by Elsevier Ltd.
This is an open access article under the CC BY license (http://creativecommons.org/licenses/by/4.0/).

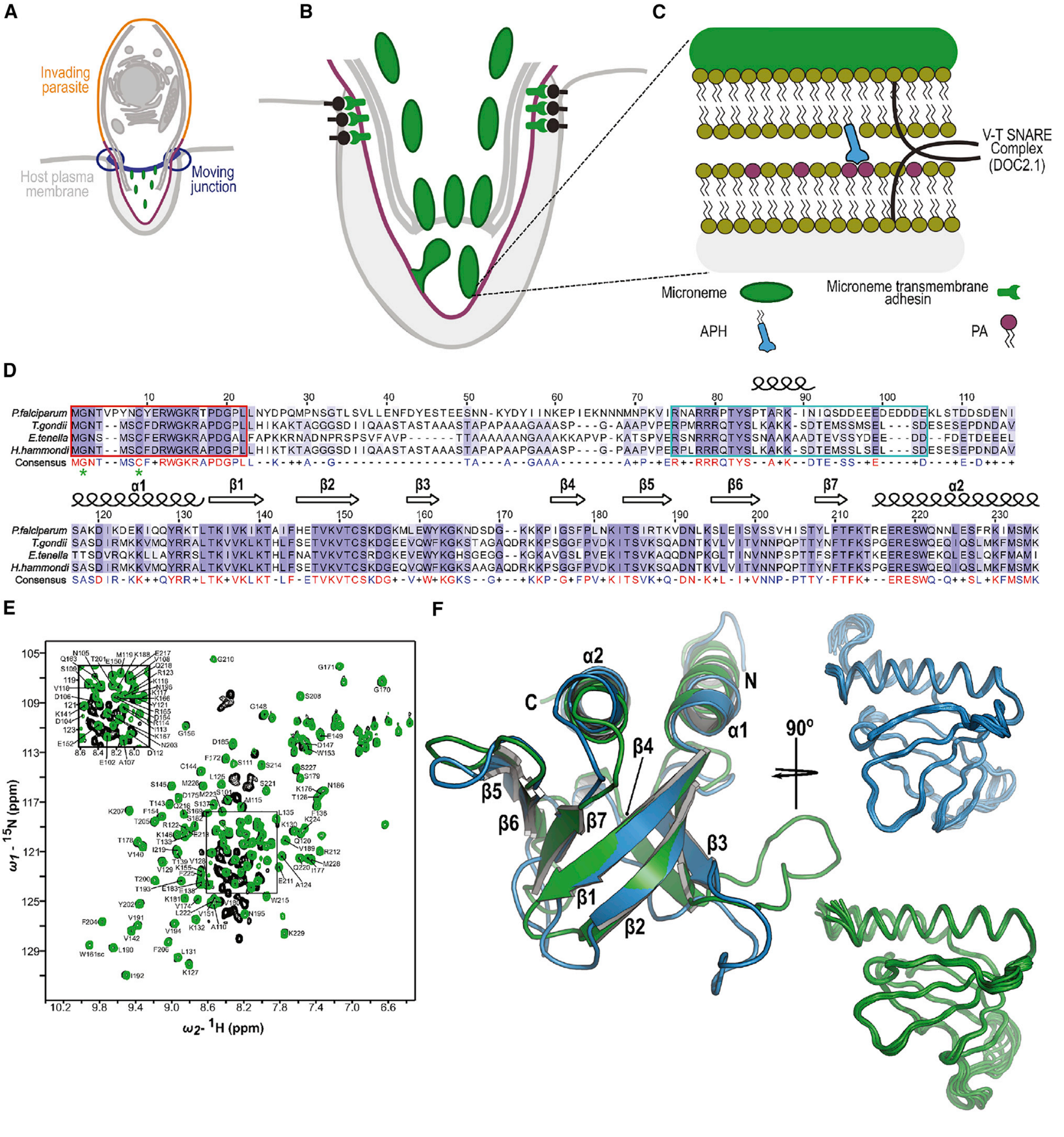

Figure 1. Structural Characterization of PfAPH$_{106-235}$ and TgAPH$_{99-229}$ Reveals the C-Terminal Region of APH (APH) Adopts a Conserved Pleckstrin Homology Domain-like Fold

(A) Schematic representation of *Toxoplasma* parasite actively invading the host cell.

(B) Schematic representation of microneme fusion with parasite plasma membrane.

(C) Close-up of fusion event. APH embedded into the microneme surface via acylation interacts with PA accumulating on the inner leaflet of the plasma membrane, facilitating microneme exocytosis. PA is represented in purple, APH in light blue, micronemes and their contents in green.

(D) Multiple sequence alignment for APH full-length sequence from different apicomplexan species. Residues are colored in a purple spectrum according to the level of sequence identity, secondary structural elements are indicated, and numbering is shown for PfAPH. The consensus sequence is given below, invariant residues are colored red, highly conserved residues are colored blue, semi-conserved sequence identity is indicated by (+), and invariant residues are indicated by (−). The highly conserved 21 N-terminal residues required for targeting to the micronemal surface are highlighted by a red box, myristoylation (G2) and palmitoylation (C7) lipid anchor sites are indicated by green asterisks. A basic region within the linker sequence containing several conserved basic residues is highlighted by a cyan box.

(legend continued on next page)

The importance of this pathway suggests that it has potential as a target for therapeutic intervention, however the finer high-resolution mechanistic details are lacking. Furthermore, the mechanism by which the APH senses changes in the local PA concentration at the plasma membrane remains unclear. Here, we fill a gap of knowledge by elucidating the atomic resolution basis of the interaction between the apicomplexan PA effector, APH, and its lipid mediator PA in a variety of contexts, and by providing new atomic details into the initiation of microneme-plasma membrane fusion prior to release of the microneme contents.

RESULTS

The Overall Atomic Structure of *T. gondii* and *P. falciparum* APH

Secondary structure predictions of APHs reveal a highly conserved mixed α/β domain at its C-terminus that is connected to the N-terminal acylation motifs via an extensive linker region (Drozdetskiy et al., 2015) (Figure 1D). Although the C-terminal half of APH possesses a structural organization consistent with a PH domain, predicted differences include an additional helical feature at its N-terminus and a shorter interstrand region between β1 and β2. Furthermore, a helical secondary structure is predicted within a charged portion of the APH linker (residues 85–91 in PfAPH) immediately upstream of the augmented PH domain. To provide further insight we compared 1D nuclear magnetic resonance (NMR) spectra for the full-length APH protein from *T. gondii* minus the acylation motif ($TgAPH_{22-229}$) with a construct representing the structured PH domain within the C-terminal 99–229 residues ($TgAPH_{99-229}$; Figure 1D). While the ordered PH domain ($TgAPH_{99-229}$) is evident from the well-dispersed NMR resonance at high and low chemical shifts (Figure S1), the NMR spectrum of $TgAPH_{22-229}$ is not consistent with the presence of an extensive disordered linker with over 20 alanine methyl resonances. It is therefore likely that many resonances for this region are broadened beyond detection due to conformational exchange on an intermediate timescale. Concurrently, only an estimated 54 out of an expected 77 backbone amide peaks belonging to the linker region, are visible in the heteronuclear single quantum coherence (HSQC) spectra for $TgAPH_{22-229}$ when compared with $TgAPH_{99-229}$ (Figure 1E). Absence of the additional linker region backbone amide resonances may be indicative of conformational exchange in this region. Interestingly, comparison between circular dichroism (CD) spectra for $TgAPH_{22-229}$ and $TgAPH_{99-229}$ indicate additional helical propensity within the N-terminal linker region (Figure S1). NMR spectra of recombinant produced PH domains from PfAPH ($PfAPH_{106-235}$) and TgAPH ($TgAPH_{99-229}$) were of excellent quality, so we determined the high-resolution solution structure of both proteins. These structures reveal an archetypal PH superfamily fold (Figure 1F; see Table 1 for structural statistics) (Lenoir et al., 2015), consisting of an open, seven-stranded β-barrel capped at one corner by a C-terminal α helix. Predicted differences to the classical PH fold, namely the β1-β2 loop and N-terminal α helix, are revealed by the experimental structures. The APH-specific N-terminal α helix packs against the C-terminus (Figure 1F), and the interstrand β1-β2 loop is much shorter and closed in APH compared with typical PH domains. These two features have potential functional implications.

The N-terminal helix of the PH domain extends to the linker region that connects to the microneme membrane anchor, and therefore this may play a role in signaling PA accumulation at the plasma membrane to the downstream membrane fusion machinery. Perhaps the most significant structural difference between APH and classic PH domains is the short β1-β2 loop, as this lies at the heart of the canonical phospholipid binding site and is usually longer and more open (Lenoir et al., 2015) (Figure S1). This striking difference indicates an altered mode of phospholipid binding for APH or a more restricted binding pocket to accommodate the small head group of PA. The similarity between $PfAPH_{106-235}$ and $TgAPH_{99-229}$ structures, and the high level of sequence conservation across the different apicomplexan species, suggest that this architecture applies to all apicomplexan APHs.

APH-PA Interface Overlaps with Canonical and Atypical Binding Surfaces

To delineate the binding site of the PA head group on the APH structure, ^{1}H-^{15}N HSQC NMR titration experiments with increasing molar ratios of short-chain PA, were performed first with ^{15}N-labeled $PfAPH_{106-235}$ due to its higher-quality spectra (Figures 2A and 2B). Chemical shift perturbation (CSP) of peaks upon addition of the PA ligand indicates a change in the chemical environment of the backbone amide group, and its likely proximity to the binding site. Small dose-dependent CSPs in fast exchange on the NMR timescale were observed for several peaks in the presence of short-chain PA. The location of these CSPs reveals a contiguous cluster around the β1 and β2 strands, suggesting that this region plays a role in recognition of PA (Figure 2C). Notably, one of the largest CSPs is observed for a lysine residue located on the β1 strand in PfAPH (K138), which represents the start of a conserved K*x*K motif. Prominent CSPs are also observed for residues that map to the β1-β2 loop region (T141 to H145 in PfAPH). Taken together, the NMR mapping of PA binding for $PfAPH_{106-235}$ reveals a contiguous surface that overlaps with canonical and atypical binding sites identified in PH domains with specificity for other phospholipids (Figure S1).

NMR studies using fast tumbling isotropic bicelles were initiated to gain further insight into the intermolecular interactions that occur in APH upon binding PA within the membrane. To limit the signal broadening of $PfAPH_{106-235}$ resonances upon the

(E) Overlay of ^{15}N-labeled $TgAPH_{22-229}$ (black) and $TgAPH_{99-229}$ (green) 2D ^{1}H-^{15}N HSQC spectra. In comparison to $TgAPH_{99-229}$, additional backbone amide peaks belonging to the linker region are visible in the $TgAPH_{22-229}$ spectrum. There is expected to be an additional 77 backbone amide peaks in this linker region, but it is estimated only ~54 peaks are visible. Residues that could be assigned in $TgAPH_{99-229}$ are labeled, sc indicates resonances could be assigned to side chains (W161sc and W215sc).

(F) Left, aligned cartoon representations of the lowest-energy structures calculated for $PfAPH_{106-235}$ (PDB: 6F24, blue) and $TgAPH_{99-229}$ (PDB: 6F8E, green), the first 11 and 10 residues are omitted from $PfAPH_{106-235}$ and $TgAPH_{99-229}$ respectively as these were shown to be disordered. Right, ensembles of the ten lowest-energy structures calculated for $PfAPH_{106-235}$ and $TgAPH_{99-229}$.

Table 1. NMR and Structural Validation Statistics for APH

NMR-Derived Restraints	PfAPH PDB: 6F24	TgAPH PDB: 6F8E
Unambiguous Nuclear Overhauser Effect		
Intra-residue	945	946
Sequential	485	494
Medium range $(\|i-j\|) \leq 4$	215	279
Long range $(\|i-j\|) > 4$	632	700
Ambiguous NOE	1,199	1,133
Dihedral angle restraints (Φ/Ψ)	230	234
Structure Statistics		
Violations		
Number of dihedral angle violations >5°	6.7 ± 1.0	2.2 ± 0.7
Number of distance constraint violations >0.5Å	0.30 ± 0.46	0.05 ± 0.22
Deviation from idealized geometry		
Bond length (Å)	0.0040 ± 0.0001	0.0040 ± 0.0001
Bond angle (°)	0.57 ± 0.01	0.56 ± 0.01
Average pairwise root-mean-square deviation r.m. SD for heavy atoms within secondary structures (Å)	0.54 ± 0.07	0.52 ± 0.05
Ramachandran plot[a]		
% In most favored positions	91.0% ± 1.0%	91.0% ± 1.0%
% In allowed regions	98.0% ± 1.0%	98.0% ± 1.0%
% In disallowed regions	2.0%	2.0% ± 1.0%

[a]Obtained from PDB NMR structure validation report.

addition of bicelles (~100 kDa size range), high long-chain length lipids (1,2-dimyristoyl-sn-glycero-3-phosphocholine [DMPC] and 1-palmitoyl-2-oleoyl-sn-glycero-3-phosphocholine [POPC] or 1-palmitoyl-2-oleoyl-sn-glycero-3-phosphate [POPA])/short-chain length lipid (1,2-diheptanoyl-sn-glycero-3-phosphocholine [DHPC]) ratios, $q = 0.33$ bicelles (where q is the relative ratio), were employed to generate smaller bicelles. Specific CSPs were observed in the ^{1}H-^{15}N HSQC spectra of PfAPH$_{106\text{-}235}$ upon the addition of bicelles with a bilayer enriched in POPA (Figure S2). Mapping these CSPs onto the structure of PfAPH$_{106\text{-}235}$ reveals clusters around W161, β1-β2 (I143, F144 and H145), and β6-β7 (I205 to T207) loop regions, consistent with those identified in short-chain PA titrations.

Finally, to delineate the PA-binding surface more precisely, we performed NMR titration experiments with PA-enriched bicelles doped with a paramagnetic lipid (5% PE-DTPA-Gd^{3+} [1,2-distearoyl-sn-glycero-3-phosphoethanolamine-N-diethylenetriaminepentaacetic acid (gadolinium salt)]) to induce enhanced transverse relaxation for residues proximal to the binding surface. Following guidelines from previous studies, the distribution of the paramagnetic lipid is expected to be random at this lipid concentration and under these conditions (Koppisetti et al., 2014; Mazhab-Jafari et al., 2015). Paramagnetic relaxation enhancements (PREs) were quantified by the reduction in signal intensities in ^{1}H-^{15}N HSQC NMR spectra, when compared with the same experiment performed in the presence of diamagnetic bicelles. Although specific PREs were observed for amides in PfAPH$_{106\text{-}235}$ (Figures 2D and 2E) that coincide with the CSP data, the PRE data also highlight the β3-β4 loop, which only showed very small chemical shifts changes in the short-chain PA titration. Interestingly, this region possesses a second conserved K*x*K motif that would likely be involved in PA recognition. Its increased prominence in the bicelle titration experiments could suggest that this motif is more specific for a PA bilayer context. The observed PA-binding site for PfAPH$_{106\text{-}235}$ was delineated in identical NMR titration experiments for TgAPH$_{99\text{-}229}$ (Figure S3).

While PIP-strip assays showed that both TgAPH and PfAPH bind specifically to PA, it was also suggested that APH may be capable of binding to PI$_{(4,5)}$P$_2$, albeit more weakly (Bullen et al., 2016). Although no other lipid specificity was suggested in these studies, we used NMR to test the possible PI$_{(4,5)}$P$_2$ binding. NMR titrations of PfAPH$_{106\text{-}235}$ with PI$_{(4,5)}$P$_2$ did not induce CSPs, confirming the absence of a specific interaction (Figure S4). Dual specificity for lipids via distinct binding sites has been reported for several PH domains (Jian et al., 2015; Lai et al., 2013; Lucas and Cho, 2011). To assess whether APH is capable of dual phosphoinositide binding or perhaps more importantly, whether PA binding enhances recognition of a second phospholipid, NMR titration experiments were performed with increasing molar ratios of short-chain PI$_{(4,5)}$P$_2$ after saturation with PA (Figure S5). As expected, the PA-specific CSPs were observed in the PfAPH$_{106\text{-}235}$ ^{1}H-^{15}N HSQC spectra, however no further CSPs occurred with PI$_{(4,5)}$P$_2$. Taken together, these data indicate that the weak PI$_{(4,5)}$P$_2$ binding observed in PIP-strip assay is likely a result of non-specific binding (Bullen et al., 2016).

APH Binds Specifically to PA-Enriched Unilamellar Vesicles

Binding experiments with recombinant APHs and short-chain PA enabled mapping of the PA head group interaction in a residue-specific manner. Notably, CSPs are small and binding to the short-chain PA is in the fast exchange regime on the NMR timescale, suggesting that the interaction, in this context, is weak (estimated to be >50 μM). To quantify the affinity and facilitate an assessment of site-directed mutants, we monitored the 1D ^{1}H NMR spectrum for PfAPH$_{106\text{-}235}$ or TgAPH$_{99\text{-}229}$ following the addition of liposomes (Ceccon et al., 2013; Mercredi et al., 2016) (Figures 3 and S5). A loss in signal intensity can be interpreted as the formation of a large, NMR-invisible complex between APH and the liposomes. Only modest signal intensity losses are observed for PfAPH$_{106\text{-}235}$ upon titration with large unilamellar vesicles (LUVs) composed solely of POPC (POPC LUVs; Figure 3A). In comparison, titration with LUVs composed of 50% POPA and 50% POPC (POPA LUVs) resulted in significant signal attenuation, with a complete loss of the PfAPH$_{106\text{-}235}$ spectrum at high liposome concentrations (Figure 3B).

Binding curves were generated from these data by integrating the upfield-shifted methyl region, plotting values against lipid concentration and fitting to a single-site binding isotherm. The apparent dissociation constant (Kd$_{app}$) for PfAPH$_{106\text{-}235}$ binding LUVs containing 50% POPA is 275 ± 13 μM, almost 5-fold lower

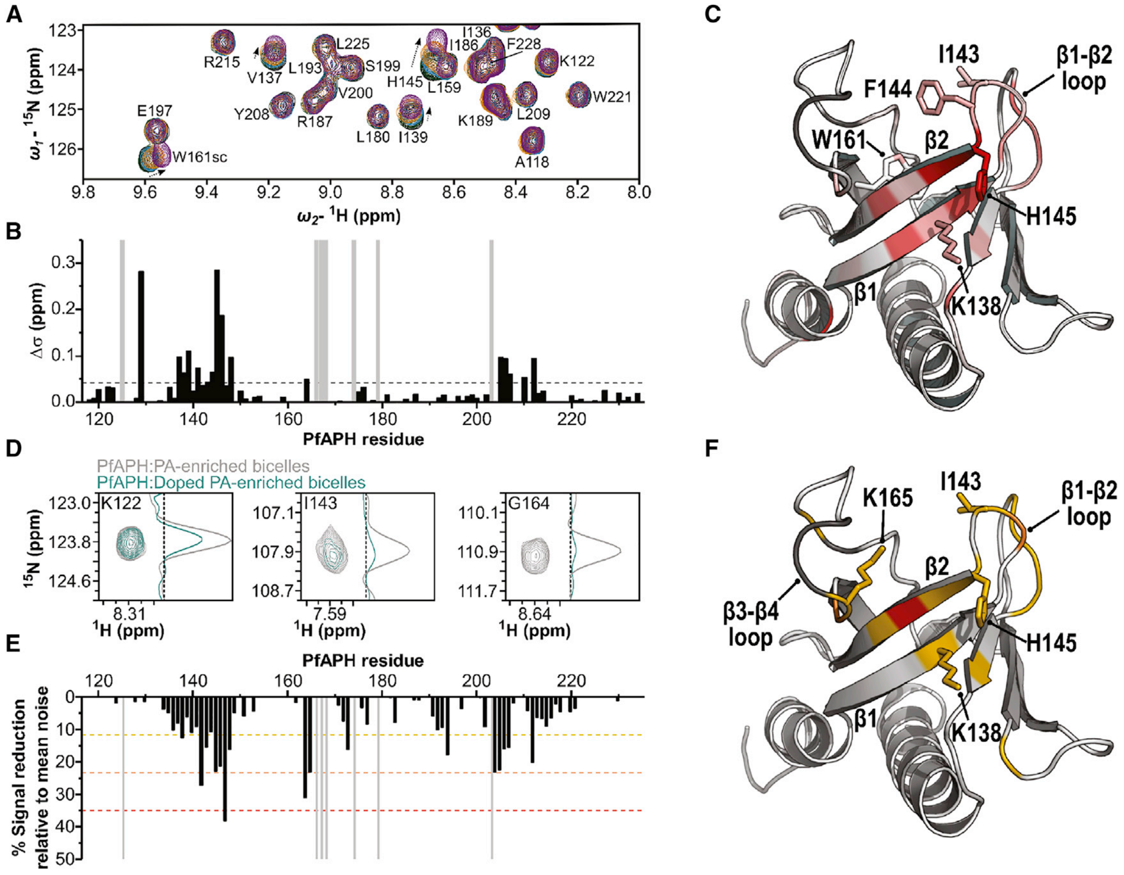

Figure 2. Mapping the APH:PA Interface

(A) Overlay of representative 2D ^{1}H-^{15}N HSQC spectra of $PfAPH_{106-235}$ recorded upon titration with increasing molar ratios of short-chain PA. HSQC spectra are colored according to the molar ratio between ^{15}N-labeled $PfAPH_{106-235}$ and short-chain PA; black 1:0, green 1:1, blue 1:3, orange 1:7, purple 1:15.

(B) Plot of CSPs observed in (A) upon titration with 15-fold molar excess of short-chain PA, versus $PfAPH_{106-235}$ sequence number. Residues that could not be assigned are indicated by a gray bar. Prominent CSPs are categorized as greater than 2σ from the mean noise (0.041 ppm), which is represented by a dotted line.

(C) CSPs mapped onto the structure of $PfAPH_{106-235}$, colored in a 20-interval red spectrum. A more intense coloring indicates a greater CSP as each interval represents 0.5σ from the mean noise. Key residues clustered around the β1/2 strands and β3-β4 loop region are labeled, unassigned residues are colored dark gray.

(D) Representative ^{1}H-^{15}N HSQC spectra and ^{15}N 1D profiles for $PfAPH_{106-235}$ recorded in the presence of PA-enriched bicelles doped with and without a paramagnetic 5% PE-DTPA-$Gd3+$ lipid. PREs and therefore proximity to the PA-binding sites are indicated by a reduction in peak intensity.

(E) Plot of peak intensity reduction observed in (D) relative to the mean noise (61.80%), which is shown as the baseline, versus $PfAPH_{106-235}$ sequence number.

(F) PREs mapped onto the structure of $PfAPH_{106-235}$, residues are colored if greater than 1σ (yellow), 2σ (orange), or 3σ (red) from the mean noise, while unassigned residues are colored dark gray.

than the Kd_{app} for POPC LUVs (1070 μM ± 33 μM), which indicates that the presence of POPA enhances the interaction between $PfAPH_{106-235}$ and LUVs (Figures 3C and 3D). To establish the general role of electrostatic interactions between LUVs and APH, titrations were repeated with LUVs in which PA was replaced with 1-palmitoyl-2-oleoyl-sn-glycero-3-phospho-L-serine (POPS), which also possesses a negatively charged head group. A value of Kd_{app} of 850 ± 13 μM was obtained for $PfAPH_{106-235}$ (Figures 3C and 3D). Although the observed trend in LUV binding affinity for $PfAPH_{106-235}$ is also borne out in identical titration experiments with $TgAPH_{99-229}$ (Figures 3E and 3F), the affinity $TgAPH_{99-229}$ shows for POPS liposomes is higher than $PfAPH_{106-235}$. The increased affinity of $PfAPH_{106-235}$ or $TgAPH_{99-229}$ for POPA LUVs over the similarly negatively charged POPS LUVs suggests interaction for PA or clusters of PA molecules is specific and not dictated by simple electrostatic attraction alone.

Twin KxK Motifs and the β1-β2 Loop Are Essential for PA Recognition

A series of alanine substitutions were generated in $PfAPH_{106-235}$ and $TgAPH_{99-229}$ (referred to with the mutation type and position hereafter) that target residues highlighted in the NMR-based PA titrations. The 1D NMR LUV assay was used to determine the influence of these mutations on PA binding. Mutation of K138 (PfAPH_K138A, Kd_{app} = 959 ± 35 μM) or K140 (PfAPH_K140A, Kd_{app} = 604 ± 29 μM) reduces $PfAPH_{106-235}$ affinity for POPA LUVs compared with wild-type (data not shown). Mutation of both lysines (PfAPH_K138A_K140A, Kd_{app} = 1,494 ± 117 μM) has a more dramatic effect on binding, reducing the affinity of

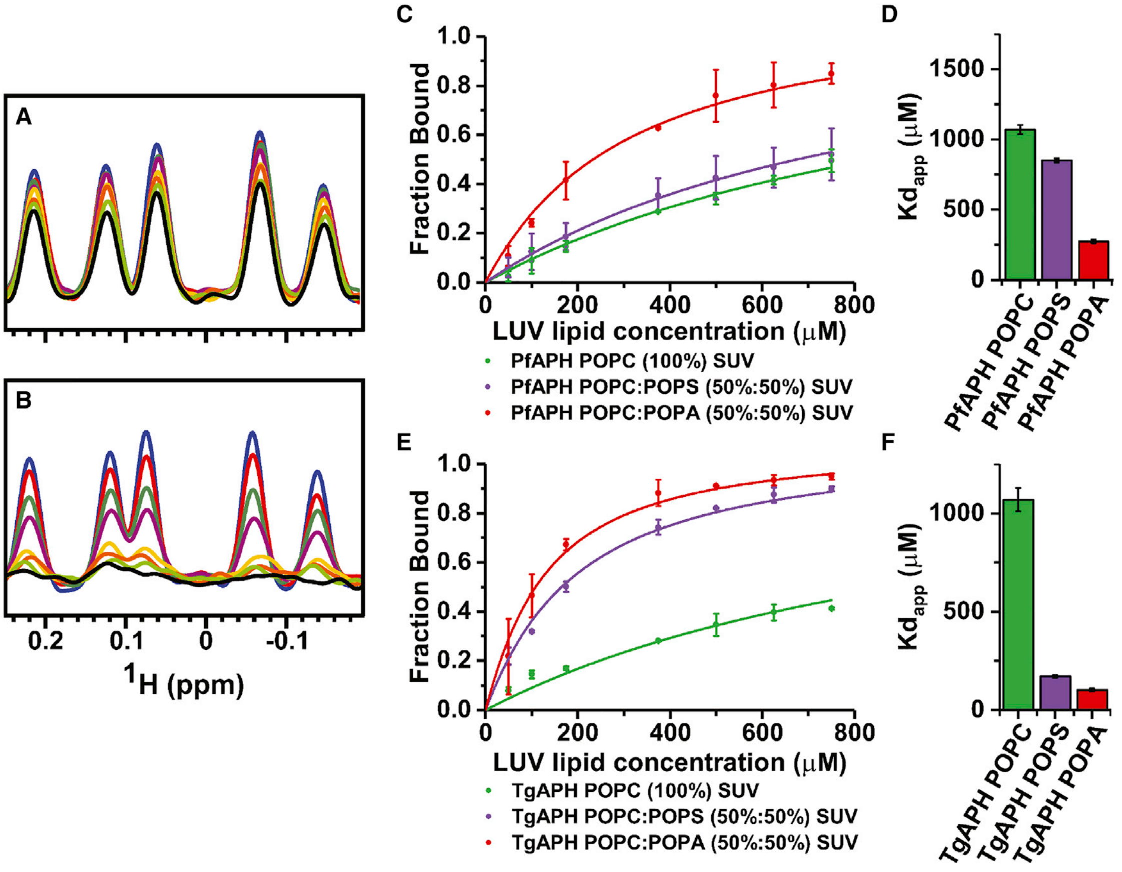

Figure 3. APH Specifically Binds PA-Enriched Membranes

(A and B) $PfAPH_{106-235}$ 1D ^{1}H NMR spectral region corresponding to the upfield-shifted methyl region (0.255 to −0.170 ppm) was monitored upon titration with increasing concentration of LUVs composed of (A) POPC (100%) or (B) POPC and POPA (50%:50%). $PfAPH_{106-235}$:LUVs molar ratios: blue, free $PfAPH_{106-235}$ in solution; red 1:2; green 1:4; purple 1:7; yellow 1:15; orange 1:20; lime 1:25; black 1:30.

(C) This region was monitored upon titration with variable LUV compositions (POPC [100%] green, POPC:POPS [50%:50%] purple, or POPC:POPA [50%:50%] red), integrated, expressed as the fraction of bound protein, and plotted against total lipid concentration to generate binding curves. Data are represented as mean ± 1σ.

(D) Apparent dissociation constants (Kd_{app}) for binding LUVs were calculated from fitting binding curves. Data are shown as mean ± 1σ for fitting curves.

(E and F) (E) and (F) are identical to (C) and (D), but for $TgAPH_{99-229}$ using the downfield-shifted amide region (9.4–6.4 ppm).

$PfAPH_{106-235}$ for POPA LUVs beyond that for wild-type binding to neutral POPC LUVs (Figures 4A and 4B). Mutation of the second conserved K*x*K motif present in the β3-β4 loop (K163 and K165 in PfAPH) has a similarly detrimental effect on PA binding, increasing Kd_{app} to 1,636 ± 54 μM for the double-mutant PfAPH_K163A_K165A (Figures 4A and 4B). $PfAPH_{106-235}$ affinity for POPA LUVs is also reduced when exposed hydrophobic side chains located at the tip of the β1-β2 loop are mutated; namely I143 and F144A (PfAPH_I143A_F144A, Kd_{app} = 1,322 ± 24 μM), and H145 (PfAPH_H145A, Kd_{app} = 499 ± 9 μM) (Figures 4A and 4B). Removal of a negative charge that disrupts the β1 strand K*x*K motif in the mutation of E146 to alanine, resulted in an increase in $PfAPH_{106-235}$ affinity for POPA LUVs (PfAPH_E146A, Kd_{app} = 155 ± 4 μM). Identical LUV titration assays with $TgAPH_{99-229}$ confirm that the PA-binding behavior is consistent with that observed for $PfAPH_{106-235}$ (Figures 4C and 4D). It is noted that compared with $PfAPH_{106-235}$, the solvent-exposed patch of basic charge present on the same face as the β3-β4 loop K*x*K motif, is extended in $TgAPH_{99-229}$ (compare Figures 4A and 4C). Electrostatic attraction between this extended basic patch and negatively charged membranes may explain why $TgAPH_{99-229}$ has a greater affinity for POPS LUVs.

Functional Characterization of Key Residues in TgAPH

In vivo validation of the importance of APH residues was determined by complementation of *T. gondii* parasites due to the availability of a strain bearing a regulatable endogenous *TgAPH* (TgAPH-iKD). The tested second copies of *TgAPH* mutants containing an internal Ty tag epitope were targeted to the non-essential uracil phosphoribosyltransferase locus (*UPRT*) (Figure 5A). TgAPH mutants were generated in the two K*x*K PA-binding motifs (TgAPH-K130A_K132A and TgAPH-K155A_K157A), the hydrophobic motif at tip of the β1-β2 loop (TgAPH-L135A_F136A) and also in the negatively charged amino acid that disrupts the β1 strand K*x*K motif (TgAPH-E138A). The charged linker region upstream of the PH domain displays significant levels of sequence conservation among apicomplexan APH (Figure 1A). A 34-amino acid sequence within this linker region was

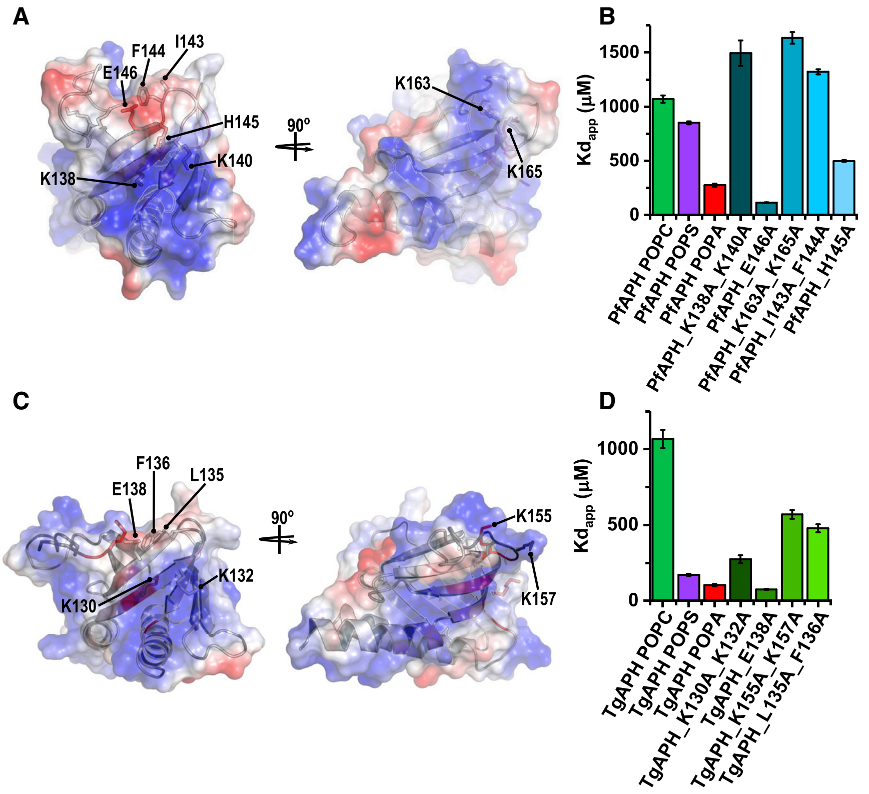

Figure 4. Conserved APH Basic Residues Mediate Binding to PA Within a Membrane Environment

(A–D) APH contains only two, highly conserved K*x*K motifs present on the β1 strand (K138 and K140 in PfAPH, K130 and K132 in TgAPH) and within the β3-β4 loop region (K163 and K165 in PfAPH, K155 and K157 in TgAPH). Coulombic colored surface representation of (A) $PfAPH_{106\text{-}235}$ and (C) $TgAPH_{99\text{-}229}$ reveals K*x*K motifs form patches of solvent-exposed basic charge. 1D NMR LUV titration experiments show that mutation of K*x*K motifs reduce (B) $PfAPH_{106\text{-}235}$ and (D) $TgAPH_{99\text{-}229}$ affinities for PA-enriched LUVs. A glutamate residue (E146 in PfAPH, E138 in TgAPH) present in the β2 strand disrupts the β1 strand K*x*K surface exposed basic charge (A and C). As measured by 1D NMR LUV titration experiments, mutation of this glutamate residue increases $PfAPH_{106\text{-}235}$ and $TgAPH_{99\text{-}229}$ affinity for PA-enriched LUVs (B and D).

subsequently deleted in TgAPH-Δlinker. To address whether features of the linker region other than its length are functionally important, this region was first deleted and then a subsequent mutation introduced a scrambled linker sequence (T*g*APH-Sc-linker). Integration and expression of these APH mutants was confirmed by genomic PCR and western blot analysis (Figure 5B). Although, the second copies of APH are expressed at lower levels compared with the endogenous or wild-type copy, the expression levels between mutants are comparable. The T*g*APH-Δlinker mutant leads to a modest decrease in protein expression, whereas the T*g*APH-Sc-linker mutant shows a striking 5- to 10-fold increase in protein levels. No changes in expression levels from the second gene copies were observed upon TgAPH-iKD depletion with anhydrotetracycline (ATc).

None of the mutants within the PH domain or the linker deletion led to defects in intracellular growth rate (±ATc), nor did they affect the lytic cycle in the absence of ATc (Figures 5C and 5D). In contrast, the scrambled linker mutant showed a marked defect in the intracellular growth rate and lytic cycle (–ATc), which is likely a result of the high expression of this APH mutant coupled with a defect in its function. All other mutants expressed at lower levels compared with wild-type APH. Upon the depletion of TgAPH-iKD, TgAPH-WT and TgAPH-E138A were capable of restoring the lytic cycle, whereas the TgAPH-K155A_K157A partially complemented the phenotype by generating small plaques. In contrast, TgAPH-K130A_K132A, TgAPH-L135A_F136A, T*g*APH-Δlinker, and T*g*APH-Sc-linker exhibited severe defects comparable to TgAPH-iKD + ATc, indicating that these mutants are non- or poorly functional variants of TgAPH (Figure 5E).

Depletion of APH also leads to a defect in microneme secretion (Bullen et al., 2016), which was partially complemented by the various mutants. TgAPH-K130A_K132A, TgAPH-L135A_F136A, and TgAPH-K155A_K157A mutants showed limited ability to secrete micronemes, with TgAPH-K130A_K132A being the most severely affected (Figure 5E), which is consistent with the plaque assay data. No microneme secretion was observed in the presence of either T*g*APH-Δlinker or T*g*APH-Sc-linker mutants, which highlight the importance of the linker sequence (Figure 5F).

The APH-Phospholipid Binding Surface Accommodates Multiple PA Head-Groups

The presence of two conserved K*x*K motifs and mutagenesis data, indicating a key role in PA binding, raises the notion that multiple phosphate head-groups may be recognized by APH. To challenge this hypothesis further, we performed coarse-grained molecular dynamics (CG-MD) simulations in which the PfAPH PH domain ($PfAPH_{106\text{-}235}$) was placed ~9 nm away from an equilibrated PC:PA lipid bilayer of varying composition from 0% to 50% PA; 3 × 5 μs simulations at each lipid composition were performed in which $PfAPH_{106\text{-}235}$ was free to diffuse and encounter the membrane (Figures 6 and S6). Similar techniques have been used to characterize the interactions of PH domains with PIP-containing membranes (Lai et al., 2013; Lumb et al., 2011; Yamamoto et al., 2016). In all simulations, $PfAPH_{106\text{-}235}$ encountered the bilayer multiple times, forming transient complexes with the membrane surface (Figure S6), the frequency of which increases with PA concentration. Mapping the contacts between $PfAPH_{106\text{-}235}$ and the lipid molecules over time at each lipid composition allowed us to probe features of binding to PA-enriched membrane.

The simulations converge upon a stable binding mode of $PfAPH_{106\text{-}235}$ on the membrane surface, which is consistent across the PA concentration range (Figures 6A and S6). In this

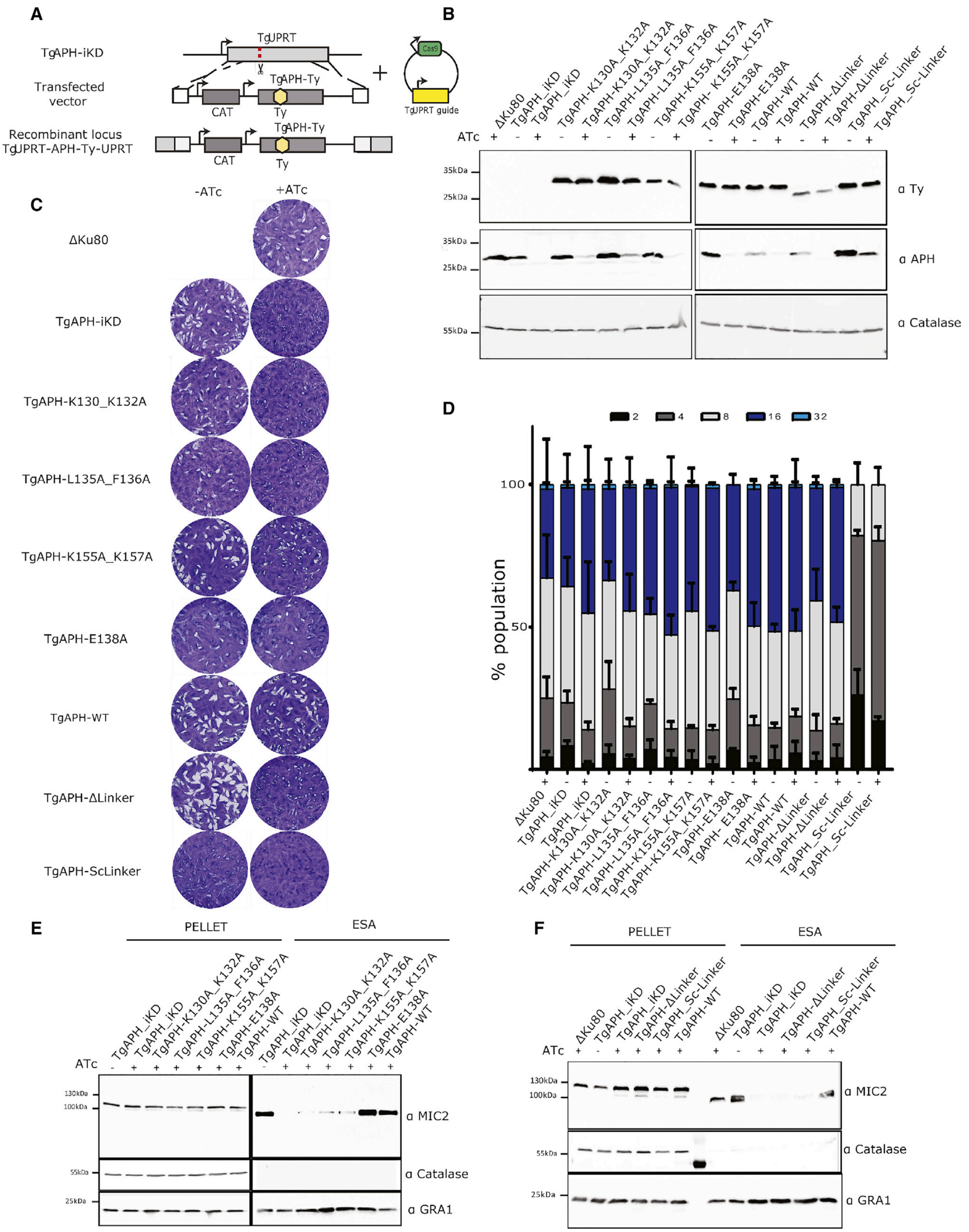

(legend on next page)

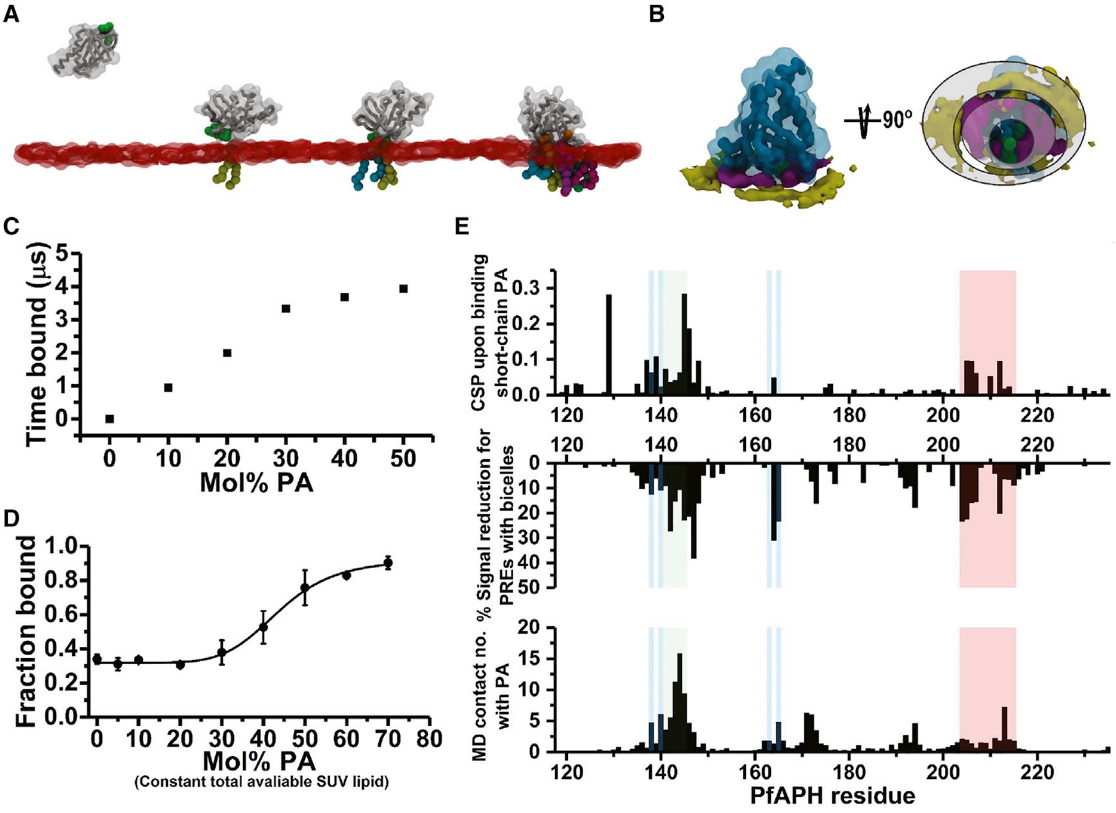

Figure 6. Coarse-Grained MD Simulation of APH Binding to PA-Enriched Membranes

(A) Snapshots from an individual binding series of $PfAPH_{106-235}$ (gray) to a 10% PA membrane. The hydrophobic residues I143-F144-H145 that become anchored in the membrane are shown as a green surface. POPA residues within 6 Å of the protein surface are shown as spheres colored individually and the lipid head-groups are shown as a transparent red surface. The recruitment of POPA following the initial association is apparent in the final panel.

(B) Average occupancy of PA (magenta) and PC (yellow) head-groups averaged over five simulations of 50% PA membranes, $PfAPH_{106-235}$ is shown in light blue. POPA is found to be preferentially in the first shell of lipids around the buried anchor residues I143-F144-H145 (green) whereas PC is found in the second annular layer. Rough lipid shell boundaries are indicated by gray-shaded circles. The protein backbone is shown as a gray trace.

(C) Relationship between average time (µs) bound to membrane and PA membrane enrichment for $PfAPH_{106-235}$ coarse-grained MD simulation (5 µs total simulation time).

(D) Binding between $PfAPH_{106-235}$ and a fixed concentration of LUVs (500 µM total available lipid) increasingly enriched with PA (Mol% PA). Hill plot analysis indicates $PfAPH_{106-235}$ binds to PA in a positively cooperative manner.

(E) Comparison between coarse-grained MD simulation and NMR experiments probing binding between $PfAPH_{106-235}$ and PA reveal three regions key to interaction with a PA-enriched membrane. Coarse-grained MD simulations indicate residues 99–110 (including β5-β6 loop) are involved in initial contact with a PA-enriched membrane (red). Hydrophobic residues located at the tip of the β1-β2 loop region (green, I143/F144) dip into the membrane. This anchoring is stabilized by electrostatic interaction between conserved charged residues including KxK motifs (blue, K138-K140 and K163-K165), and PA head-groups.

bound state, the hydrophobic residues I143-F144-H145 in the β1-β2 loop penetrate the membrane leaflet surface, whereas the K*x*K motifs accommodate multiple, negatively charged PA head-groups. The APH membrane contact points identified from independent MD simulations are consistent with the binding interface highlighted in NMR mapping experiments (Figure 6E). At all concentrations of PA, upon binding of the protein, POPA molecules are recruited to the protein, with up to six POPA lipids present at the interface in 10% PA membranes (Figure S6). PA lipids cluster tightly in the first "shell" around the anchoring loop, with PC lipids displaced to form the second layer or "shell" around the protein (Figures 6B and S6). Furthermore, PA forms small clusters of dimers and trimers within the CG membrane (Figure S6).

Taken together, the experimental observation of at least two PA-binding sites on APH suggests that binding PA-enriched

Figure 5. *In Vivo* Functional and Mutagenesis Studies of APH

(A) Schematic representation of APH-Ty mutant generation.

(B) Western blot analysis of endogenous and second copy *Tg*APH ± ATc 48 hr. Catalase provides a loading control.

(C) Plaque assay on human foreskin fibroblast monolayer 7 days ± ATc.

(D) Intracellular growth assays at 24 hr ± ATc treatment, with 24-hr pre-treatment. Data are presented as mean ± 1σ.

(E and F) Microneme secretion assay of mutants in the PH domain (E) and linker region (F). Extracellular secreted antigen (ESA) MIC2 was compared with parental strain ± ATc 48 hr. Catalase represents a loading control for parasite number and lysis, GRA1 represents a control for constitutive secretion.

membranes may be cooperative. Initial encounter with a PA head group and β1-β2 loop insertion in the membrane leaflet could in turn enhance the affinity for a second PA molecule. To test this, we performed NMR binding experiments with $PfAPH_{106-235}$, in which the proportions of PA within the LUV bilayer was increased, while the total concentration of LUVs was kept constant (Figure 6D). The fraction of bound APH increased sharply only when PA levels in the LUV were above 40% and began to plateau above 70% (Hill constant, $n = 6.77 \pm 0.72$; goodness of fit $R^2 > 0.99$). Bilayer binding rates from individual MD simulations can be calculated for membranes with increasing PA concentration (Figure 6C). Although the curve is shifted to lower concentrations, likely due to the limitations of a CG-MD model, the dependence of time bound on the PA concentration echoes the sigmoidal curve of the NMR binding data (Figure 6C).

DISCUSSION

PH domains are ubiquitous in signal transduction pathways. The vast majority of the PH domains characterized to date bind phosphatidylinositol phosphates or inositol phosphate head-groups and subsequently target proteins to a specific endomembrane compartment (Lemmon, 2008). The structures of $TgAPH_{99-229}$ and $PfAPH_{106-235}$ reveal deviations from the canonical PH domain fold, with an N-terminal helix connected to the APH-microneme linker and a much shorter β1-β2 loop (Figure 1). The binding surface of the PA head-groups also encompasses both canonical and atypical binding sites of typical PH domains that target phosphatidylinositol/inositol phosphates (Figure 2). Canonical binding sites comprise a basic sequence within the β1-β2 loop, usually Kx_n(K/R)*x*R that coordinates phosphates from PIP ligands. PH domains lacking this motif often use the opposite face of the β1-β2 strands and the intervening loop, which has been termed the atypical binding site and is observed for the β-spectrin and ArhGap9 PH domains (Ceccarelli et al., 2007; Yamamoto et al., 2016). Although the Kx_n(K/R)*x*R motif is not present in any of the APH sequences, they harbor two conserved, but separated K*x*K motifs that are important for binding of PA-enriched membranes. The lysine side chains of the first motif (K138-K140 in PfAPH or K130-K132 in TgAPH) project toward the atypical binding surface on the upper side of the β1-β2 region. The second motif (K163-K165 in PfAPH or K155-K157 in TgAPH) delineates one edge of the canonical binding site, which is capped by the shorter and closed β1-β2 loop. Two further basic sequences are conserved in APH, namely RRR within the linker (R78-R79-R80 in PfAPH or R73-R74-R75 in TgAPH) and K/RxK in the β3-β4 loop (K171-K173 in PfAPH or R165-K167 in TgAPH), and these may play minor roles in membrane binding.

The true nature of PH domain interactions with membranes is far more complex than single phospholipid recognition. Recent structural studies on PH domains revealed that PIPs can bind to both canonical and atypical sites simultaneously (Jian et al., 2015; Vonkova et al., 2015), and often in a cooperative manner; for example, by the PH domain from the Arf GAP (Vonkova et al., 2015) and ASAP1 (Jian et al., 2015). Despite these advances, reports on PH PA binding are sparse and no structural insight is currently available. Mutagenesis of the nucleotide-exchange factor Son of Sevenless (Sos) implicated a role for two positively charged residues from an extended β3-β4 loop in PA binding and subsequent Ras activation. These observations raise the possibility that another phospholipid interaction may play a role in APH membrane engagement, i.e., in addition to PA, and the relationship could be cooperative. Our studies do not support additional phospholipid specificity for APH, but instead reveal a high selectivity for PA that is driven through cooperative binding to more than one PA lipid molecule. Two major PA-binding sites exist within the APH domain, which are represented by two conserved K*x*K motifs, with the first lying on the atypical face within β1 and the other at the end of β3. These motifs lie in distinct locations on the APH structure and are juxtaposed to the well-established canonical and atypical binding sites of PH domains. A similar behavior has been reported for the kindlin-3 and Brag2 PH domains, which are able to accommodate multiple PIP lipid head-groups (Karandur et al., 2017; Ni et al., 2017). The β1-β2 loop is also important for the membrane association of APH, with the surface exposed, bulky hydrophobic side chains from this region (I143-F144-H145 in PfAPH), inserting into the lipid bilayer. It is conceivable that membrane insertion of the β1-β2 loop occurs after initial encounter of a K*x*K motif with a PA head group and subsequent conformation change could facilitate increased binding to the bilayer. Similar conclusions have been postulated from structural and dynamic studies of PH domains from ACAP1 and Grp1 (Lumb et al., 2011; Pang et al., 2014). It is widely recognized that some PA-binding proteins respond to negative curvature stress (Putta et al., 2016). This property together with the multiple contacts of APH with PA head-groups and insertion of the hydrophobic loop may provide a mechanism to sense increased local PA concentration and subsequent negative curvature.

The presence of a basic region linker sequence between the PH domain and the microneme N-terminal anchor (residues R75-K89 in PfAPH and R70-K84 in TgAPH) is worth noting. The *in vivo* complementation data confirm that the APH linker is critical and plays a key functional role in triggering microneme secretion. The conserved basic "Rx_2RRRx_8RK" would be influenced by the proximity of the negatively charged membrane surface in membrane-bound APH. It is tempting to speculate that the PH domain together with the basic APH linker interact with the negatively charged bilayer and further stabilize binding to the PA-enriched plasma membrane. It is worth noting that in tandem BAR-PH domain proteins, such as ACAP1 or the ArfGAPs (Frost et al., 2009), an additional basic surface on the helical BAR domain enhances interaction of the PH domains with the membrane surface and induces curvature. Upon sensing PA at the parasitic plasma membrane, APH tethers the micronemal membrane in close proximity. This function is somewhat reminiscent of Num1, a protein that tethers mitochondria to the plasma membrane in budding yeast through a bipartite interaction (Ping et al., 2016). Num1 C-terminal PH domain binds $PI_{(4,5)}P_2$ at plasma membrane, whereas the N-terminal coiled coil domain preferentially binds cardiolipin at the mitochondrial outer membrane via basic residues. Similarities between the domain architecture of Num1 and APH may also suggest that the N-terminal linker region in APH plays a role in membrane binding.

The association of APH with the plasma membrane leading to the engagement of microneme fusion and exocytosis is poorly understood. Presumably, these events are not independent

and are connected either via an APH-induced molecular signal or a direct interaction. Indeed, many PA-binding effectors are targeted through cooperative binding with additional protein co-factors (Lemmon, 2008). For example, Opi1 binds and senses changes in PA in the ER of yeast by binding to the ER protein Scs2 in addition to PA (Loewen et al., 2004). The engagement of APH molecules with the PA-enriched regions of the plasma membrane could provide a stable molecular scaffold for the subsequent recruitment of membrane fusion machinery, e.g., SNARE-like DOC2 proteins (Farrell et al., 2012).

STAR★METHODS

Detailed methods are provided in the online version of this paper and include the following:

- KEY RESOURCES TABLE
- CONTACT FOR REAGENT AND RESOURCE SHARING
- EXPERIMENTAL MODEL AND SUBJECT DETAILS
  - HFF Cell Culture
  - *Toxoplasma gondii* Cell Culture
- METHOD DETAILS
  - PfAPH and TgAPH Cloning, Expression and Purification for Structural Studies
  - $PfAPH_{106-235}$ and $TgAPH_{99-229}$ Short-Chain Phosphatidic Acid and $PI_{(4,5)}P_2$ ^1H-^{15}N HSQC Titration Experiments
  - Large Unilamellar Vesicle (LUV) Preparation
  - 1D ^1H-NMR LUV Titration Experiments
  - Bicelle Preparation
  - $PfAPH_{106-235}$ and $TgAPH_{99-229}$ Bicelle HSQC Titration and PRE Experiments
  - $PfAPH_{106-235}$ and $TgAPH_{99-229}$ NMR Resonance Assignment and Structure Calculation
  - Circular Dichroism
  - Cloning of DNA Constructs for *In-Vivo* Studies
  - Parasite Transfection and Selection of Stable Transfectants
  - Western Blot Analysis
  - Microneme Secretion Assay
  - Plaque Assay
  - *T. gondii* Growth Assay
  - Immunofluorescence Assay
  - Molecular Dynamics Simulations
- QUANTIFICATION AND STATISTICAL ANALYSIS
- DATA AND SOFTWARE AVAILABILITY
  - HSQC Titration Analysis
  - PRE Analysis
  - 1D ^1H-NMR LUV Titration Analysis
  - Circular Diochroism

SUPPLEMENTAL INFORMATION

Supplemental Information includes six figures and three tables and can be found with this article online at https://doi.org/10.1016/j.str.2018.05.001.

ACKNOWLEDGMENTS

The authors would like to thank the Wellcome Trust (PhD program; equipment grant no. 085464 awarded to S.M.) and The Leverhulme Trust (grant RPG-2018-107). This research was supported by the Swiss National Science Foundation 310030B_166678 (to D.S.-F.). D.J.D is supported by IZRJZ3_164183 (to Dr. Karine Frenal).

AUTHOR CONTRIBUTIONS

N.D., D.J.D., and S.L.R designed and conducted experiments, wrote the paper, and constructed figures. P.M.H., T.B., and S.B. conducted experiments. B.L. contributed to solving NMR structures. D.S.-F. and S.M. designed experiments, wrote the paper, and secured funding.

DECLARATION OF INTERESTS

The authors declare no competing financial interests.

Received: February 8, 2018
Revised: March 26, 2018
Accepted: May 8, 2018
Published: June 14, 2018

SUPPORTING CITATIONS

The following references appear in the Supplemental Information: Kim et al., 1993.

REFERENCES

Bhatt, S., Weiss, D.J., Cameron, E., Bisanzio, D., Mappin, B., Dalrymple, U., Battle, K.E., Moyes, C.L., Henry, A., Eckhoff, P.A., et al. (2015). The effect of malaria control on *Plasmodium falciparum* in Africa between 2000 and 2015. Nature *526*, 207–211.

Brochet, M., Collins, M.O., Smith, T.K., Thompson, E., Sebastian, S., Volkmann, K., Schwach, F., Chappell, L., Gomes, A.R., Berriman, M., et al. (2014). Phosphoinositide metabolism links cGMP-dependent protein kinase G to essential Ca2+ signals at key decision points in the life cycle of malaria parasites. PLoS Biol. *12*, e1001806.

Bullen, H.E., Jia, Y., Yamaryo-Botte, Y., Bisio, H., Zhang, O., Jemelin, N.K., Marq, J.B., Carruthers, V., Botte, C.Y., and Soldati-Favre, D. (2016). Phosphatidic acid-mediated signaling regulates microneme secretion in toxoplasma. Cell Host Microbe *19*, 349–360.

Bussi, G., Donadio, D., and Parrinello, M. (2007). Canonical sampling through velocity rescaling. J. Chem. Phys. *126*, 014101.

Carruthers, V.B., and Sibley, L.D. (1999). Mobilization of intracellular calcium stimulates microneme discharge in *Toxoplasma gondii*. Mol. Microbiol. *31*, 421–428.

Ceccarelli, D.F.J., Blasutig, I.M., Goudreault, M., Li, Z., Ruston, J., Pawson, T., and Sicheri, F. (2007). Non-canonical interaction of phosphoinositides with pleckstrin homology domains of Tiam1 and ArhGAP9. J. Biol. Chem. *282*, 13864–13874.

Ceccon, A., D'Onofrio, M., Zanzoni, S., Longo, D.L., Aime, S., Molinari, H., and Assfalg, M. (2013). NMR investigation of the equilibrium partitioning of a water-soluble bile salt protein carrier to phospholipid vesicles. Proteins *81*, 1776–1791.

Cowman, A.F., and Crabb, B.S. (2006). Invasion of red blood cells by malaria parasites. Cell *124*, 755–766.

de Jong, D.H., Singh, G., Bennett, W.F., Arnarez, C., Wassenaar, T.A., Schafer, L.V., Periole, X., Tieleman, D.P., and Marrink, S.J. (2013). Improved parameters for the Martini Coarse-grained protein force field. J. Chem. Theory Comput. *9*, 687–697.

Drozdetskiy, A., Cole, C., Procter, J., and Barton, G.J. (2015). JPred4: a protein secondary structure prediction server. Nucleic Acids Res. *43*, W389–W394.

Farrell, A., Thirugnanam, S., Lorestani, A., Dvorin, J.D., Eidell, K.P., Ferguson, D.J., Anderson-White, B.R., Duraisingh, M.T., Marth, G.T., and Gubbels, M.J. (2012). A DOC2 protein identified by mutational profiling is essential for apicomplexan parasite exocytosis. Science *335*, 218–221.

Fox, B.A., Ristuccia, J.G., Gigley, J.P., and Bzik, D.J. (2009). Efficient gene replacements in *Toxoplasma gondii* strains deficient for nonhomologous end joining. Eukaryot. Cell *8*, 520–529.

Frost, A., Unger, V.M., and De Camilli, P. (2009). The BAR domain superfamily: membrane-molding macromolecules. Cell *137*, 191–196.

Hedger, G., Rouse, S.L., Domanski, J., Chavent, M., Koldso, H., and Sansom, M.S. (2016). Lipid-loving ANTs: molecular simulations of cardiolipin interactions and the organization of the adenine nucleotide translocase in model mitochondrial membranes. Biochemistry *55*, 6238–6249.

Hess, B., Kutzner, C., van der Spoel, D., and Lindahl, E. (2008). GROMACS 4: algorithms for highly efficient, load-balanced, and scalable molecular simulation. J. Chem. Theory Comput. *4*, 435–447.

Humphrey, W., Dalke, A., and Schulten, K. (1996). VMD: visual molecular dynamics. J. Mol. Graph. *14*, 33–38, 27-38.

Huynh, M.H., and Carruthers, V.B. (2009). Tagging of endogenous genes in a *Toxoplasma gondii* strain lacking Ku80. Eukaryot. Cell *8*, 530–539.

Jacot, D., Tosetti, N., Pires, I., Stock, J., Graindorge, A., Hung, Y.F., Han, H., Tewari, R., Kursula, I., and Soldati-Favre, D. (2016). An apicomplexan actin-binding protein serves as a connector and lipid sensor to coordinate motility and invasion. Cell Host Microbe *20*, 731–743.

Jean, S., Zapata-Jenks, M.A., Farley, J.M., Tracy, E., and Mayer, D.C. (2014). *Plasmodium falciparum* double C2 domain protein, PfDOC2, binds to calcium when associated with membranes. Exp. Parasitol. *144*, 91–95.

Jian, X., Tang, W.K., Zhai, P., Roy, N.S., Luo, R., Gruschus, J.M., Yohe, M.E., Chen, P.W., Li, Y., Byrd, R.A., et al. (2015). Molecular basis for cooperative binding of anionic phospholipids to the PH domain of the Arf GAP ASAP1. Structure *23*, 1977–1988.

Jimah, J.R., Salinas, N.D., Sala-Rabanal, M., Jones, N.G., Sibley, L.D., Nichols, C.G., Schlesinger, P.H., and Tolia, N.H. (2016). Malaria parasite CelTOS targets the inner leaflet of cell membranes for pore-dependent disruption. Elife *5*, e20621.

Jung, Y.S., and Zweckstetter, M. (2004). Mars – robust automatic backbone assignment of proteins. J. Biomol. NMR *30*, 11–23.

Karandur, D., Nawrotek, A., Kuriyan, J., and Cherfils, J. (2017). Multiple interactions between an Arf/GEF complex and charged lipids determine activation kinetics on the membrane. Proc. Natl. Acad. Sci. USA *114*, 11416–11421.

Kim, Y., and Schneider, K.A. (2013). Evolution of drug resistance in malaria parasite populations. Nat. Educ. Knowl *4*, 6.

Kim, K., Soldati, D., and Boothroyd, J.C. (1993). Gene replacement in *Toxoplasma gondii* with chloramphenicol acetyltransferase as selectable marker. Science *262*, 911–914.

Koldso, H., Shorthouse, D., Helie, J., and Sansom, M.S. (2014). Lipid clustering correlates with membrane curvature as revealed by molecular simulations of complex lipid bilayers. PLoS Comput. Biol. *10*, e1003911.

Koppisetti, R.K., Fulcher, Y.G., Jurkevich, A., Prior, S.H., Xu, J., Lenoir, M., Overduin, M., and Van Doren, S.R. (2014). Ambidextrous binding of cell and membrane bilayers by soluble matrix metalloproteinase-12. Nat. Commun. *5*, 5552.

Lai, C.-L., Srivastava, A., Pilling, C., Chase, A.R., Falke, J.J., and Voth, G.A. (2013). Molecular mechanism of membrane binding of the GRP1 PH domain. J. Mol. Biol. *425*, 3073–3090.

Lemmon, M.A. (2008). Membrane recognition by phospholipid-binding domains. Nat. Rev. Mol. Cell Biol. *9*, 99–111.

Lenoir, M., Kufareva, I., Abagyan, R., and Overduin, M. (2015). Membrane and protein interactions of the pleckstrin homology domain superfamily. Membranes *5*, 646–663.

Loewen, C.J., Gaspar, M.L., Jesch, S.A., Delon, C., Ktistakis, N.T., Henry, S.A., and Levine, T.P. (2004). Phospholipid metabolism regulated by a transcription factor sensing phosphatidic acid. Science *304*, 1644–1647.

Lucas, N., and Cho, W. (2011). Phosphatidylserine binding is essential for plasma membrane recruitment and signaling function of 3-phosphoinositide-dependent kinase-1. J. Biol. Chem. *286*, 41265–41272.

Lumb, C.N., He, J., Xue, Y., Stansfeld, P.J., Stahelin, R.V., Kutateladze, T.G., and Sansom, M.S.P. (2011). Biophysical and computational studies of membrane penetration by the GRP1 pleckstrin homology domain. Structure *19*, 1338–1346.

Mazhab-Jafari, M.T., Marshall, C.B., Smith, M.J., Gasmi-Seabrook, G.M.C., Stathopulos, P.B., Inagaki, F., Kay, L.E., Neel, B.G., and Ikura, M. (2015). Oncogenic and RASopathy-associated K-RAS mutations relieve membrane-dependent occlusion of the effector-binding site. Proc. Natl. Acad. Sci. USA *112*, 6625.

McGuffin, L.J., Bryson, K., and Jones, D.T. (2000). The PSIPRED protein structure prediction server. Bioinformatics *16*, 404–405.

Meissner, M., Brecht, S., Bujard, H., and Soldati, D. (2001). Modulation of myosin A expression by a newly established tetracycline repressor-based inducible system in *Toxoplasma gondii*. Nucleic Acids Res. *29*, E115.

Mercredi, P.Y., Bucca, N., Loeliger, B., Gaines, C.R., Mehta, M., Bhargava, P., Tedbury, P.R., Charlier, L., Floquet, N., Muriaux, D., et al. (2016). Structural and molecular determinants of membrane binding by the HIV-1 matrix protein. J. Mol. Biol. *428*, 1637–1655.

Moudy, R., Manning, T.J., and Beckers, C.J. (2001). The loss of cytoplasmic potassium upon host cell breakdown triggers egress of *Toxoplasma gondii*. J. Biol. Chem. *276*, 41492–41501.

Ni, T., Kalli, A.C., Naughton, F.B., Yates, L.A., Naneh, O., Kozorog, M., Anderluh, G., Sansom, M.S.P., and Gilbert, R.J.C. (2017). Structure and lipid-binding properties of the kindlin-3 pleckstrin homology domain. Biochem. J. *474*, 539–556.

Oostenbrink, C., Villa, A., Mark, A.E., and van Gunsteren, W.F. (2004). A biomolecular force field based on the free enthalpy of hydration and solvation: the GROMOS force-field parameter sets 53A5 and 53A6. J. Comput. Chem. *25*, 1656–1676.

Pang, X., Fan, J., Zhang, Y., Zhang, K., Gao, B., Ma, J., Li, J., Deng, Y., Zhou, Q., Egelman, E.H., et al. (2014). A PH domain in ACAP1 possesses key features of the BAR domain in promoting membrane curvature. Dev. Cell *31*, 73–86.

Ping, H.A., Kraft, L.M., Chen, W., Nilles, A.E., and Lackner, L.L. (2016). Num1 anchors mitochondria to the plasma membrane via two domains with different lipid binding specificities. J. Cell Biol. *213*, 513–524.

Pirahmadi, S., Zakeri, S., Mehrizi, A.A., and Djadid, N.D. (2018). Analysis of genetic diversity and population structure of gene encoding cell-traversal protein for ookinetes and sporozoites (CelTOS) vaccine candidate antigen in global *Plasmodium falciparum* populations. Infect. Genet. Evol. *59*, 113–125.

Putta, P., Rankenberg, J., Korver, R.A., van Wijk, R., Munnik, T., Testerink, C., and Kooijman, E.E. (2016). Phosphatidic acid binding proteins display differential binding as a function of membrane curvature stress and chemical properties. Biochim. Biophys. Acta *1858*, 2709–2716.

Rieping, W., Habeck, M., Bardiaux, B., Bernard, A., Malliavin, T.E., and Nilges, M. (2007). ARIA2: automated NOE assignment and data integration in NMR structure calculation. Bioinformatics *23*, 381–382.

Robert-Gangneux, F., and Darde, M.L. (2012). Epidemiology of and diagnostic strategies for toxoplasmosis. Clin. Microbiol. Rev. *25*, 264–296.

Roiko, M.S., and Carruthers, V.B. (2013). Functional dissection of *Toxoplasma gondii* perforin-like protein 1 reveals a dual domain mode of membrane binding for cytolysis and parasite egress. J. Biol. Chem. *288*, 8712–8725.

Santos, J.M., and Soldati-Favre, D. (2011). Invasion factors are coupled to key signalling events leading to the establishment of infection in apicomplexan parasites. Cell. Microbiol. *13*, 787–796.

Scott, K.A., Bond, P.J., Ivetac, A., Chetwynd, A.P., Khalid, S., and Sansom, M.S. (2008). Coarse-grained MD simulations of membrane protein-bilayer self-assembly. Structure *16*, 621–630.

Shen, Y., Delaglio, F., Cornilescu, G., and Bax, A. (2009). TALOS+: a hybrid method for predicting protein backbone torsion angles from NMR chemical shifts. J. Biomol. NMR *44*, 213–223.

Shen, B., Brown, K.M., Lee, T.D., and Sibley, L.D. (2014). Efficient gene disruption in diverse strains of *Toxoplasma gondii* using CRISPR/CAS9. MBio *5*, e01114.

Singh, S., and Chitnis, C.E. (2012). Signalling mechanisms involved in apical organelle discharge during host cell invasion by apicomplexan parasites. Microbes Infect. *14*, 820–824.

Soldati, D., and Boothroyd, J.C. (1993). Transient transfection and expression in the obligate intracellular parasite *Toxoplasma gondii*. Science *260*, 349–352.

Vonkova, I., Saliba, A.-E., Deghou, S., Anand, K., Ceschia, S., Doerks, T., Galih, A., Kugler, K.G., Maeda, K., Rybin, V., et al. (2015). Lipid cooperativity as a general membrane-recruitment principle for PH domains. Cell Rep. *12*, 1519–1530.

Williamson, M.P. (2013). Using chemical shift perturbation to characterise ligand binding. Prog. NMR Spectrosc. *73*, 1–16.

Yamamoto, E., Kalli, A.C., Yasuoka, K., and Sansom, M.S.P. (2016). Interactions of Pleckstrin homology domains with membranes: adding back the bilayer via high-throughput molecular dynamics. Structure *24*, 1421–1431.

STAR★METHODS

KEY RESOURCES TABLE

REAGENT or RESOURCE	SOURCE	IDENTIFIER
Antibodies		
α-APH	The Soldati-Favre Lab	N/A
α-Ty	The Soldati-Favre Lab	N/A
α-Catalase	The Soldati-Favre Lab	N/A
α-MIC2	The Carruthers Lab	N/A
α-GAP45	The Soldati-Favre Lab	N/A
α-GRA3	The Dubremetz Lab	N/A
Peroxidase conjugated goat α-mouse/rabbit	ThermoFisher	Cat# 62-6520, Cat# 31460
Alexa Fluor 680 conjugated goat α-rabbit	ThermoFisher	Cat# A-21109
Alexa Fluor 488 conjugated goat α-mouse	ThermoFisher	Cat# A-11001
Alexa Fluor 594 conjugated goat α-rabbit	ThermoFisher	Cat# R37117
Bacterial and Virus Strains		
XL10 Gold	Stratagene	Cat# 200315
E. coli DH5α	NEB	Cat# C2987I
E. coli BL21 (DE3)	NEB	Cat# C2527I
Deposited Data		
Solution structure of PfAPH$_{106-235}$	This paper	PDB: 6F24
Solution structure of TgAPH$_{99-229}$	This paper	PDB: 6F8E
Experimental Models: Organism/Strains		
Human foreskin fibroblasts (HFFs)	Igcstandards	Cat# ATTC-112Sk
Toxoplasma gondii: RHΔKu80	(Fox et al., 2009; Huynh and Carruthers, 2009)	N/A
E. coli: pNIC28a-Bsa4_*PfAPH*$_{106-235}$	This paper	N/A
E. coli: pNIC28a-Bsa4_*TgAPH*$_{99-229}$	This paper	N/A
E. coli: pNIC28a-Bsa4_*TgAPH*$_{22-229}$	This paper	N/A
Oligonucleotides		
Primers used for APH expression and functional characterisation see Table S2	This paper	N/A
Recombinant DNA		
Plasmids used for expression of APH and functional studies see Table S1.	This paper	N/A
Software and Algorithms		
Sequence Manipulation Suite: Shuffle Protein	GenScript	https://www.genscript.com/sms2/shuffle_protein.html
PSI-PRED	(McGuffin et al., 2000)	http://bioinf.cs.ucl.ac.uk/psipred/
Pymol	Version 1.3 (DeLano Scientific LLC/ Schrödinger)	https://pymol.org/
Prism	Version 7, GraphPad Software	https://www.graphpad.com
OriginPro	2017 version, OriginLab	https://www.originlab.com/index.aspx?go=PRODUCTS/Origin
Topspin	Version 3.5, Bruker	https://www.bruker.com/products/mr/nmr/nmr-software/nmr-software/topspin/overview.html
MARS	(Jung and Zweckstetter, 2004)	http://www3.mpibpc.mpg.de/groups/zweckstetter/_links/software_mars.htm
TALOS+	(Shen et al., 2009)	https://spin.niddk.nih.gov/bax/software/TALOS/

(*Continued on next page*)

Continued

REAGENT or RESOURCE	SOURCE	IDENTIFIER
NMRView	NMRviewJ/In house version	http://www.onemoonscientific.com/nmrviewj/
Aria/CNS	Versions 2.3 and 1.1(Rieping et al., 2007)	http://aria.pasteur.fr/
Gromacs	Versions 4.6 and 5, (Hess et al., 2008)	www.gromacs.org

CONTACT FOR REAGENT AND RESOURCE SHARING

Further information and requests for resources and reagents should be directed to and will be fulfilled by the Lead Contact, Steve Matthews (s.j.matthews@imperial.ac.uk).

EXPERIMENTAL MODEL AND SUBJECT DETAILS

HFF Cell Culture

Human foreskin fibroblasts (HFFs) were grown at 37°C, 5% CO_2 in Dulbecco's Modified Eagle's Medium (DMEM; GIBCO, Invitrogen) supplemented with 2 mM glutamine, 5% foetal calf serum and 25 μg/ml gentamicin.

Toxoplasma gondii Cell Culture

RHΔKu80 were grown at 37°C, 5%CO_2 in confluent human foreskin fibroblasts (HFFs) maintained in DMEM, supplemented with 2 mM glutamine, 5% foetal calf serum and 25 μg/ml gentamicin. Tet-inducible gene expression was regulated with 1 μg/ml anhydrotetracycline (ATc) (Meissner et al., 2001).

Escherichia coli *DH5α Cell Culture*

Transformed DH5α strains were grown in LB media or plated onto LB agar supplemented with 50μg/ml Kanamycin (Sigma), and grown at 37°C.

Escherichia coli *BL21 Cell Culture*

Transformed BL21 strains were grown at 37°C in either LB media or M9 media supplemented with $^{15}NH_4Cl$ and/or ^{13}C-glucose until OD_{600} reached 0.8 units. Media was supplemented with 50μg/ml Kanamycin (Sigma). Expression was induced at 18°C by the addition of 0.5mM IPTG (Sigma) for $PfAPH_{106\text{-}235}$/$TgAPH_{99\text{-}229}$, or 0.25mM for $TgAPH_{22\text{-}229}$.

METHOD DETAILS

PfAPH and TgAPH Cloning, Expression and Purification for Structural Studies

Based on secondary structure prediction (PSI-PRED), the sequence corresponding to the C-terminal pleckstrin-homology domain was amplified from full length, codon optimised *PfAPH* and *TgAPH* genes (*PfAPH* and *TgAPH*) and cloned into an pNIC28a-Bsa4 vector containing an TEV cleavable N-terminal-$(His)_6$ tag fusion, using LIC methods, to generate pNIC28a-Bsa4_$PfAPH_{106\text{-}235}$ and pNIC28a-Bsa4_$TgAPH_{99\text{-}229}$ (see Table S1). $TgAPH_{22\text{-}229}$ was amplified from the full length codon optimised gene, excluding the conserved acylation site corresponding to the first N-terminal 21 residues. $PfAPH_{106\text{-}235}$ mutants, K138A_K140A/I143A_F144A/H145A/E146A and K163A_K165A, and $TgAPH_{99\text{-}229}$ mutants K130A_K132A/L135A_F136A/E138A and K155A_K157A were generated using Q5 site-directed mutagenesis kits (NEB) using pNIC28a-Bsa4_$PfAPH_{106\text{-}235}$ and pNIC28a-Bsa4_$TgAPH_{99\text{-}229}$ vectors as respective templates (see Table S2). DH5α *E.coli* (NEB) were used for cloning.

Vectors were transformed into an *E.coli* BL21 strain (NEB) and grown as stated in experimental models. Cells were lysed and clarified by centrifugation at 17,000rpm for 35mins. Supernatants were initially purified by nickel-affinity chromatography followed by TEV cleavage during overnight dialysis to remove the N-terminal-$(His)_6$ fusion tag. Cleaved protein was further purified by gel filtration using a Superdex-75 column (GE healthcare) pre-equilibrated in 10mM HEPES, 0.3M NaCl, 2mM TCEP, pH 6.5 ($PfAPH_{106\text{-}235}$), 50mM HEPES, 150mM NaCl, 2mM TCEP, pH 7 ($TgAPH_{99\text{-}229}$) buffer, or 10mM HEPES, 300mM NaCl, 2mM TCEP, pH7 ($TgAPH_{22\text{-}229}$). Expression and purification of PfAPH and TgAPH mutants followed the same procedure as wild-type protein. Like wild-type protein, the folding status of each mutant was verified by 1D ^1H-NMR.

$PfAPH_{106\text{-}235}$ and $TgAPH_{99\text{-}229}$ Short-Chain Phosphatidic Acid and $PI_{(4,5)}P_2$ ^1H-^{15}N HSQC Titration Experiments

550μl NMR samples were prepared with purified ^{15}N-labelled protein (250 μM final concentration) and D_20 added (10% v/v). Short-chain PA (1,2-dihexanoyl-sn-glycero-3-phosphate, Avanti lipids) and $PI_{(4,5)}P_2$ (1,2-dioctanoyl-sn-glycero-3-phospho-(1'-myo-inositol-4',5'-bisphosphate, Avanti lipids) was initially dissolved in chloroform. Chloroform was removed by evaporation under a stream of N_2 to leave a lipid film, which was left to dry overnight. Dried lipid was rehydrated with gel filtration buffer to generate a concentrated lipid stock (40mM). 2D ^1H-^{15}N HSQC spectra were recorded for protein alone and protein titrated with increasing molar ratios of short-PA or $PI_{(4,5)}P_2$ from the concentrated stock.

Large Unilamellar Vesicle (LUV) Preparation

POPA (1-palmitoyl-2-oleoyl-sn-glycero-3-phosphate), POPS (1-palmitoyl-2-oleoyl-sn-glycero-3-phospho-L-serine) and POPC (1-palmitoyl-2-oleoyl-sn-glycero-3-phosphocholine) were obtained commercially (Avanti) as chloroform dissolved lipids. Volumes of lipids were pipetted into glass vials,chloroform removed by evaporation under a stream of N_2 to leave a lipid film, and residual chloroform removed by desiccation overnight. Dried lipids were re-suspended in gel filtration buffer through shaking (1500rpm) at room temperature for 2hrs to generate a cloudy solution. To form LUVs, the re-suspension was sonicated using a probe tip sonicator until transparent and then centrifuged at 17,000rpm to removed titanium debris and large or multilamellar vesicles. LUVs were prepared at an 8mM total lipid concentration and used within 48hrs of preparation. LUVs had a typical hydrodynamic diameter of between 80-100 nm , which was measured by dynamic light scattering (Malvern, Zetasizer Nano S DLS analyser).

1D ^{1}H-NMR LUV Titration Experiments

550μl NMR samples were prepared with purified protein (50μM final concentration) and D_20 (10% v/v). For each titration at the specified large unilamellar vesicle (LUV) concentration, a LUV preparation was added to the NMR sample from a concentration stock (8mM), and mixed (see Table S3). 1D ^{1}H-NMR spectra were recorded after each titration and overlaid using Topspin 3.5 software (Bruker).

Bicelle Preparation

Bicelle lipids DMPC (1,2-dimyristoyl-sn-glycero-3-phosphocholine), DHPC (1,2-diheptanoyl-sn-glycero-3-phosphocholine), POPA (1-palmitoyl-2-oleoyl-sn-glycero-3-phosphate), POPC (1-palmitoyl-2-oleoyl-sn-glycero-3-phosphocholine) and PE-DTPA-Gd^{3+} (1,2-distearoyl-sn-glycero-3-phosphoethanolamine-N-diethylenetriaminepentaacetic acid (gadolinium salt)) were obtained commercially (Avanti) as chloroform dissolved lipids. Volumes of long chain lipids (DMPC, POPA, POPC or PE-DTPA-Gd^{3+}, depending on bicelle composition) and short-chain lipid (DHPC) were separately pipetted into glass vials. Chloroform was removed by evaporation under a stream of N_2 to leave a lipid film, and residual chloroform removed by desiccation overnight. Long-chain lipids were re-suspended in gel filtration buffer through shaking (1500rpm) at room temperature for 2hrs to generate a cloudy solution. Large unilamellar vesicles (LUVs) were generated from re-suspended long-chain lipids using methods previously described. To prepare isotropic bicelles with a q value of 0.33 (q = [Long-chain lipids]/[DHPC]), dried DHPC was re-suspended with long-chain lipid LUVs through vortexing, and the solution subjected to 10 freeze-thaw cycles between liquid nitrogen and a 60°C water bath. Bicelles were generated using a 40mM total lipid concentration, and in accordance with previously published results (Koppisetti et al., 2014), had an average hydrodynamic diameter of ~10nm which was measured using dynamic-light scattering (Malvern, Zetasizer Nano S DLS analyser). The long-chain lipid composition of POPA enriched bicelles contained varying molar ratios of DMPC and POPA, whilst POPC enriched bicelles contained a 50%:50% DMPC:POPC long-chained lipid composition. Bicelles doped with PE-DTPA-Gd^{3+} had a 50%:45%:5% POPA:POPC:PE-DTPA-Gd^{3+} long-chained lipid composition. Bicelles were used within 24 hrs of preparation.

$PfAPH_{106-235}$ and $TgAPH_{99-229}$ Bicelle HSQC Titration and PRE Experiments

Prepared bicelles (30mM final total lipid concentration) were diluted with ^{15}N-labelled protein (50μM final concentration) and D_20 (10% v/v), to generate a 550μl NMR sample. Separate NMR samples were prepared for each bicelle composition, including samples containing bicelles increasingly enriched with POPA. 2D ^{1}H-^{15}N HSQC spectra were recorded for protein alone and in the presence of bicelles. Spectra were overlaid and combined chemical shift-perturbations values were calculated. For studies with paramagnetic probes, separate NMR samples were prepared with PA-enriched bicelles doped with and without PE-DPTA-Gd^{3+}. Separate 2D ^{1}H-^{15}N HSQC spectra were recorded for protein in the presence of non-doped or doped bicelles.

$PfAPH_{106-235}$ and $TgAPH_{99-229}$ NMR Resonance Assignment and Structure Calculation

500μl samples of purified ^{15}N/^{13}C-$PfAPH_{106-235}$ (700μM) or ^{15}N/^{15}C-$TgAPH_{99-229}$ (830μM) were prepared and D20 added (10% v/v). All NMR spectra were acquired at 298K on Bruker Avance-III DRX 800 and Avance-III 600 spectrometers. An initial 1D ^{1}H NMR spectra and 2D ^{1}H-^{15}N HSQC spectra were acquired prior to and between acquisition of 3D-NMR experiments used for backbone and side chain assignment, to assess protein folding and the quality of the sample. Triple resonance HNCA, HNCACB, HNCO and HN(CO)CA spectra were recorded and analysed to obtain backbone assignments. Linking assigned backbone chemical shifts was performed automatically using MARS (Jung and Zweckstetter, 2004) which incorporates PSI-PRED secondary structure prediction (McGuffin et al., 2000). Triple resonance HBHA(CO)NH, H(CCO)NH and CC(CO)NH and HCCH-TOCSY spectra were recorded for use in side-chain chemical shift assignment. ^{15}N-NOESY and ^{13}C-NOESY spectra were recorded, peaks picked, and peak files used as distance restraints in structural calculation. Chemical shift assignment and analysis was performed using an in-house version of NMRview. Dihedral angles were calculated using TALOS+ (Shen et al., 2009) and used as restraints in structural calculations. Automatic NOE assignment and structural calculation were performed using Aria 2.3/CNS 1.1 software (Rieping et al., 2007). A set of 100 structures were calculated in the final iteration and the 10 lowest-energy structures were refined in water. Structure ensembles for $PfAPH_{106-235}$ and $TgAPH_{99-229}$ have been deposited in the PDB under accession codes 6F24 and 6F8E, respectively. The medoid structure from ensembles is represented in figures using PyMOL.

Circular Dichroism

Purified $TgAPH_{99-229}$ and $TgAPH_{22-229}$ were dialysed into 10mM HEPES, 150mM NaF, 1mM TCEP, pH7 buffer overnight at 4°C, and then diluted with 10mM HEPES, 150mM NaF, pH7 buffer to 40μM and 30μM respectively for use in circular dichroism (CD). 200μl samples were loaded into a quartz 100-QS cuvette with a 1mm path length, and CD was performed on a Chirascan circular dichroism spectrometer (Applied Photophysics) at 20°C, wavelength 200 to 260nm, 5s scan length per point, 5 repeats.

Cloning of DNA Constructs for *In-Vivo* Studies

All amplifications were performed with either KOD polymerase (Novagen) or Q5 polymerase (New England Biolabs). RNA was isolated using TRIzol extraction. Total cDNA was generated by RT-PCR using the Superscript II reverse transcriptase (Invitrogen) according to manufacturer's protocol. Primers used are listed in the Key Resources Table above.

APH gRNA/Cas9 Vector

Specific gRNA/Cas9 vector used for the generation of APH-iKD was made using the Q5 site-directed mutagenesis kit (New England Biolabs) with 6326-4883 and pSAG1::CAS9-GFP-U6::sgUPRT as a template (Shen et al., 2014).

APH Complementation

pT8-N21-Ty-APH-BleO (Bullen et al., 2016) was digested with EcoRI-PacI and ligated into 5'UPRT-pT8-MycGFPPfMyoAtail-Ty-3'UPRT (Jacot et al., 2016), pTub5-CAT was then digested with SpeI-ApaI and inserted into the intermediate plasmid generating 5'UPRT-CAT-pT8-N21-Ty-APH -3'UPRT. The modified APH variants were generated via Q5 mutagenesis of 5'UPRT-CAT-pT8-N21-Ty-APH-3'UPRT. The constructs and primers were used as follows, 5'UPRT-CAT-pT8-N21-Ty-APH-K130A+K132A-3'UPRT(6339-6529), 5'UPRT-CAT-pT8-N21-Ty-APH-L135A+F136A-3'UPRT (6341-6342), 5'UPRT-CAT-pT8-N21-Ty-APH-K155A+K157A-3'UPRT (6343-6344), 5'UPRT-CAT-pT8-N21-Ty-APH-E138A-3'UPRT (7327-7368), 5'UPRT-CAT-pT8-N21-Ty-APH-Δ-linker-3'UPRT(6423-6424). 5'UPRT-CAT-pT8-N21-Ty-APH-Sc-linker-3'UPRT was generated via triple ligation of amplicons (2170-7400) ClaI-XmaI, (7399-4749) XmaI-NotI and inserted into 5'UPRT-CAT-pT8-N21-Ty-APH -3'UPRT ClaI-NotI. The scrambled amino acid sequence was generated using the Shuffle protein program - Genscript.

Parasite Transfection and Selection of Stable Transfectants

T. gondii tachyzoites were transfected by electroporation as previously described (Soldati and Boothroyd, 1993). TgAPH-iKD strain was generated via transfection of RHΔKu80 (here referred as ΔKu80) (Fox et al., 2009; Huynh and Carruthers, 2009) with 30μg of pSAG1::CAS9-GFP-U6::sgAPH vector along with purified KOD PCR amplicon using primers 6324-6325 with iKD-GAC-DHFR (Jacot et al., 2016) as the template. Resistant parasites were selected using pyrimethamine (1 μg/ml). TgAPH-iKD strain was transfected with 5μg pSAG1::CAS9-GFP-U6::sgUPRT and 30μg of one of the TgAPH complementation plasmids (digested KpnI-NotI), refer to list above. Resistant parasites were selected using chloramphenicol (20μM). Parasites were cloned by limiting dilution in 96 well plates and plates and analysed for the integration and expression of the transgenes by PCR and Western blot, respectively.

Western Blot Analysis

3-4mL of parasites were lysed in 80μL RIPA buffer (150 mM NaCl, 1% Triton X-100, 0.5% deoxycholate, 0.1% SDS, 50mM Tris pH 7.5) using standard procedures and suspended to 120μL final volume with SDS–PAGE loading buffer (50mM Tris-HCl pH 6.8, 10% glycerol, 2mM EDTA, 2% SDS, 0.05% bromophenol blue, 100mM DTT) under reducing conditions. This suspension was subjected to 5min boiling at 95°C and two sonication cycles. SDS-PAGE was performed using standard methods, between 5-15μL of parasites were loaded per well. Separated proteins were transferred to nitrocellulose membranes and probed with appropriate antibodies in 5-10mL of 5% non-fat milk powder in 0.05%Tween20-PBS. Bound secondary peroxidase conjugated antibodies were visualized using either the ECL system (GE healthcare) or SuperSignal (Pierce).

Microneme Secretion Assay

3-4mL of freshly egressed parasites ± ATc 48hrs were resuspended in equal volume intracellular (IC) buffer (5mM NaCl, 142mM KCl, 1mM MgCl2, 2mM EGTA, 5.6mM glucose, 25mM HEPES, pH to 7.2 with KOH) prior to pelleting at 1050 rpm, 10 minutes. Pellets were subsequently washed in 500μL IC buffer and re-pelleted. Pellets were resuspended in 100μL of serum-free media and incubated with 2% EtOH for 30min at 37°C. Parasites were pelleted at 1000g, 5min at 4°C. The supernatant was subsequently transferred to new Eppendorf tubes and re-pelleted at 2000g, 5min at 4°C. Final supernatant (ESA - excreted secreted antigens) and pellet fractions were resuspended in 120μL SDS sample buffer final volume and subjected to 5min boiling at 95°C and two sonication cycles, prior to immunoblotting.

Plaque Assay

HFF monolayers were infected with 100μL of serially diluted parasites (1/100, 1/1000 and 1/10000) and allowed to develop for 7 days ± ATc. Plaques were fixed in 200μL of 4% paraformaldehyde, 0.05% glutaraldehyde (PAF-Glu), 10 minutes quenched in 600μL of 0.1M glycine-PBS and subsequently stained with 200μL Crystal Violet (Sigma-Aldrich), 10min. Data are representative of three independent biological experiments.

T. gondii Growth Assay

20μL of freshly egressed parasites ± ATc 24hrs were inoculated onto HFF coated coverslips. 24hrs post-infection the parasites were fixed with 200μL PAF-Glu for 20min, quenched in 600μL 0.1M glycine-PBS. Growth was assessed via immunofluorescence assay staining for both GAP45 (1/10000) and GRA3 (1/2000). 100 vacuoles were counted for three independent experiments. Data presented is mean value ± SD of experiments.

Immunofluorescence Assay

Previously fixed cells were permeabilized 20min in 100μL 0.2%Triton-PBS, blocked for 20min in 100μL 2%BSA-PBS. 100μL of primary antibodies (diluted as required in PBS) were incubated for 1hr, washed 3 times in 500μL PBS, followed by a 1hr incubation of 100μL of secondary antibodies and washed as previously. Coverslips were mounted onto slides with 3-5μL DAPI-Fluromount G (SouthernBiotech).

Molecular Dynamics Simulations

Simulations were performed using gromacs 4.6 and gromacs 5 (www.gromacs.org) with GPU acceleration (Hess et al., 2008). The lowest-energy PfAPH NMR model was simulated using the GROMOS56a3 force field (Oostenbrink et al., 2004) in 0.15 M NaCl for 100 ns. Multiple frames from the final 75 ns of this simulation were used to generate MARTINI version 2.2 (http://md.chem.rug.nl/) (de Jong et al., 2013) coarse-grained PfAPH parameters with the martinize.py script, using an elastic network for structured regions with a 1 nm cutoff and a force constant of 500 kJ mol^{-1} nm^{-2}. A 300-lipid POPC membrane was generated by self-assembly(Scott et al., 2008) and individual lipids from each leaflet were randomly converted to POPA in order to generate symmetric mixed PC:PA bilayers of the following compositions: 0%, 10%, 20%, 30%, 40% and 50% POPA (Koldso et al., 2014). The protein was then centered at 9 nm from the membrane centre-of-mass and randomly rotated in x, y, and z dimensions to generate 5 separate starting points for each lipid composition. Simulations were performed at 310 K using the V-rescale algorithm and 1 tau using the Parrinello-Rahman barostat (Bussi et al., 2007) with semiisotropic coupling. Visualisation used Pymol (http://pymol.org) and VMD (Humphrey et al., 1996). Lipid density isosurfaces of phosphate particles in the reference frame of the protein were generated using the Volmap plugin of VMD. Lipid contacts were calculated between each residue of the protein and the phosphate headgroup particles of POPA and POPC using a cutoff of 1.0 nm. Lipid contact analysis was performed as described elsewhere (Hedger et al., 2016) using scripts from Heidi Koldsoe (D.E.Shaw Research).

QUANTIFICATION AND STATISTICAL ANALYSIS

The coordinates of the final ensembles of $PfAPH_{106\text{-}235}$ and $TgAPH_{99\text{-}229}$ structures are deposited at the Protein Data Bank Europe (https://www.ebi.ac.uk/pdbe/) under the accession codes 6F24 and 6F8E respectively. $PfAPH_{106\text{-}235}$ and $TgAPH_{99\text{-}229}$ assigned chemical shifts are also deposited at the Biological Magnetic Resonance Bank (http://www.bmrb.wisc.edu/) under the accession numbers 34202 and 34216 respectively.

DATA AND SOFTWARE AVAILABILITY

HSQC Titration Analysis

Spectra were overlaid and chemical shifts measured for assigned backbone resonances using NMRview software. All combined chemical shift perturbation values were calculated using $((\Delta^1H \text{ chemical shift})^2 + (0.2\Delta^{15}N \text{ chemical shift}))^{1/2}$ (Williamson, 2013). Mean noise was calculated using an iterative method (Williamson, 2013).

PRE Analysis

Peak intensities for assigned backbone amide resonances were measured using NMRview software. Signal reduction was obtained from (I^*/I^o), where I^* and I^o are equal to peak intensities in the presence of doped and non-doped bicelles respectively, and expressed as a percentage. Signal reduction was subtracted from mean noise to obtain relative signal reduction.

1D ^{1}H-NMR LUV Titration Analysis

Using Topspin 3.5 software (Bruker), peaks in the region corresponding to amide (9.5 to 6.4ppm) and aliphatic methyl (0.255 to -0.175ppm) groups were integrated for $TgAPH_{99\text{-}229}$ and $PfAPH_{106\text{-}235}$1D ^{1}H-NMR spectra respectively, to exclude resonances from lipids and unstructured protein regions. The fraction of bound protein is expressed as $1\text{-}I/I_0$, where I is the integral of protein NMR signal for a given total available lipid concentration (AL_C) and I_0 is the integral of protein NMR signal when $AL_C = 0$ (protein alone). Total available lipid is calculated as half the total lipid concentration added to account for the inaccessible lipid present in the LUV inner leaflet. OriginPro software was used to plot fraction of bound protein against AL_C and fit non-linear binding isotherms to estimate apparent dissociation constants (Kd_{app}) according to (Ceccon et al., 2013):

$$Fraction\ bound = \frac{Bmax\left(([P_c] + [AL_C] + Kd_{app}) - \sqrt{\left(([P_c] + [AL_C] + Kd_{app})^2 - 4[P_c][AL_C]\right)}\right)}{2[P_c]}$$

P_C represents the total protein concentration and B_{max} is a fixed constant. Titrations with varying LUV compositions were replicated in triplicate whilst titrations with POPA enriched LUVs for $TgAPH_{99-229}$ or $PfAPH_{106-235}$ mutants were replicated in duplicate. Error bars for binding curves represent1σ from the mean for replicates, whilst error bars for calculated Kd_{app} represent 1σ from the mean for fitting binding curves.

Circular Diochroism

From 5 repeats, spectra were averaged, corrected for baseline contributions, and the net spectra smoothed with a Savitsky–Golay filter (window 2).

Structure, Volume *26*

Supplemental Information

Structural Basis of Phosphatidic Acid

Sensing by APH in Apicomplexan Parasites

Nick Darvill, David J. Dubois, Sarah L. Rouse, Pierre-Mehdi Hammoudi, Tom Blake, Stefi Benjamin, Bing Liu, Dominique Soldati-Favre, and Steve Matthews

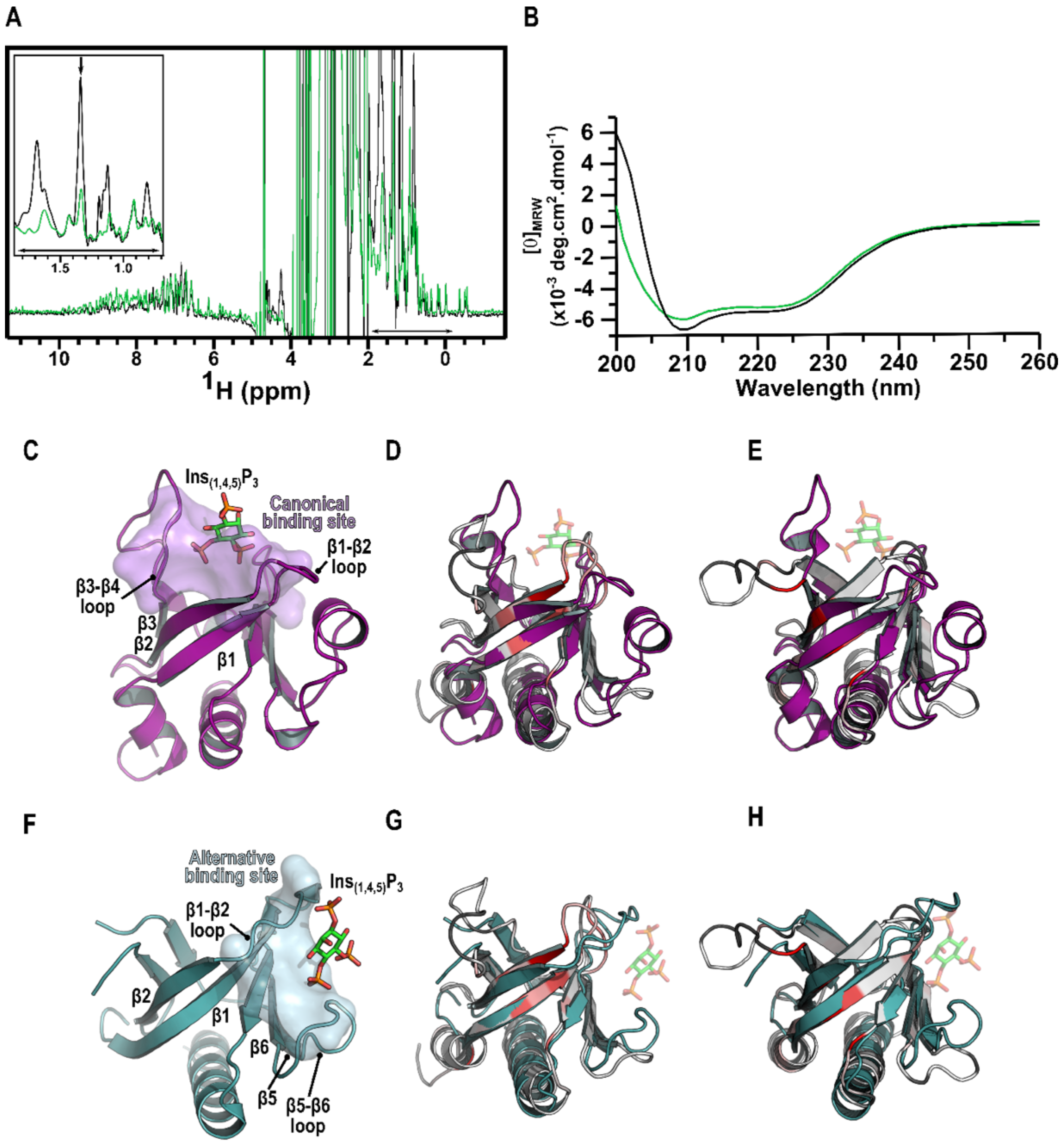

SI Figure S1 **(Related to Figure 1 and 2). Comparison between $TgAPH_{22-229}$ and $TgAPH_{99-229}$ HSQC spectra, and APH compared to canonical and alternative binding PH domains.** A) Overlay of 1D NMR spectra for TgAPH99-229 (green) and TgAPH22-229 (black). Inset, enlarged 1D spectral region highlights the peak corresponding to Ala methyl groups (indicated by a black arrow) that in TgAPH22-229, has a lower intensity than expected. B) CD spectra for TgAPH99-229 (green) and TgAPH22-229 (black). C) Cartoon representation of the crystal structure of the PH domain from R.norvegicus PLC_δ1 (PDB ID 1MAI) bound to inositol 1,4,5-triphosphate ($Ins_{(1,4,5)}P_3$) as an example of a canonical binding PH domain. The canonical binding site is represented as a purple semi-transparent surface. D) and E) PLC_δ1 PH domain aligned to $PfAPH_{106-235}$ and $TgAPH_{99-229}$ respectively with bound $Ins_{(1,4,5)}P_3$ shown for reference to the canonical binding site. F) Cartoon representation of the crystal structure of the PH domain from human ArhGAP9 (PDB ID 2p0D) bound to $Ins_{(1,4,5)}P_3$ as an example of an alternative binding PH domain. The alternative binding site is represented as a cyan semi-transparent surface. G) and H) ArhGAP9 PH domain aligned to $PfAPH_{106-235}$ and $TgAPH_{99-229}$ respectively with bound $Ins_{(1,4,5)}P_3$ shown for reference to the alternative binding site.

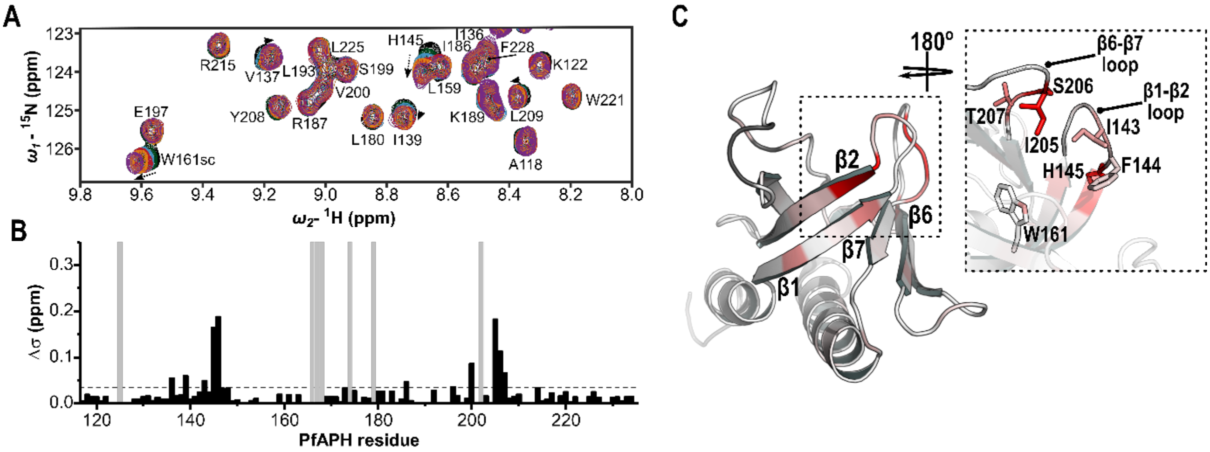

SI Figure S2 **(Related to Figure 2). PfAPH$_{106\text{-}235}$ titration with PA enriched bicelles.** A) Representative data showing overlay of ^{15}N-labelled PfAPH$_{106\text{-}235}$ 2D ^{1}H-^{15}N HSQC spectra in the presence of increasingly PA enriched bicelles with the following DHPC:DMPC:POPA composition: *black*, 75%:25%:25%; *green*, 75%:24%:1%; *blue*, 75%:18.75%:6.25%; *red*, 75%:16.25%:8.75%; *orange*, 75%:12.5%:12.5%; *purple*, 75%:10%:15%. B) Plot of CSPs observed in A) upon titration with DHPC:POPC:POPA (75%:12.5%:12.5%) bicelles, versus PfAPH$_{106\text{-}235}$ sequence number, residues that could not be assigned are indicated by a grey bar. Prominent CSP's are categorised as greater than 2σ's from the mean noise (0.035ppm), which is represented by a dotted line. C) CSPs mapped onto the structure of PfAPH$_{106\text{-}235}$, coloured in a 20 interval *red* spectrum. A more intense colouring indicates a greater CSP as each interval represents 0.5σ from the mean noise. Key residues clustered around the β1-β2 and β6-β7 loops are labelled, unassigned residues are coloured *dark grey*.

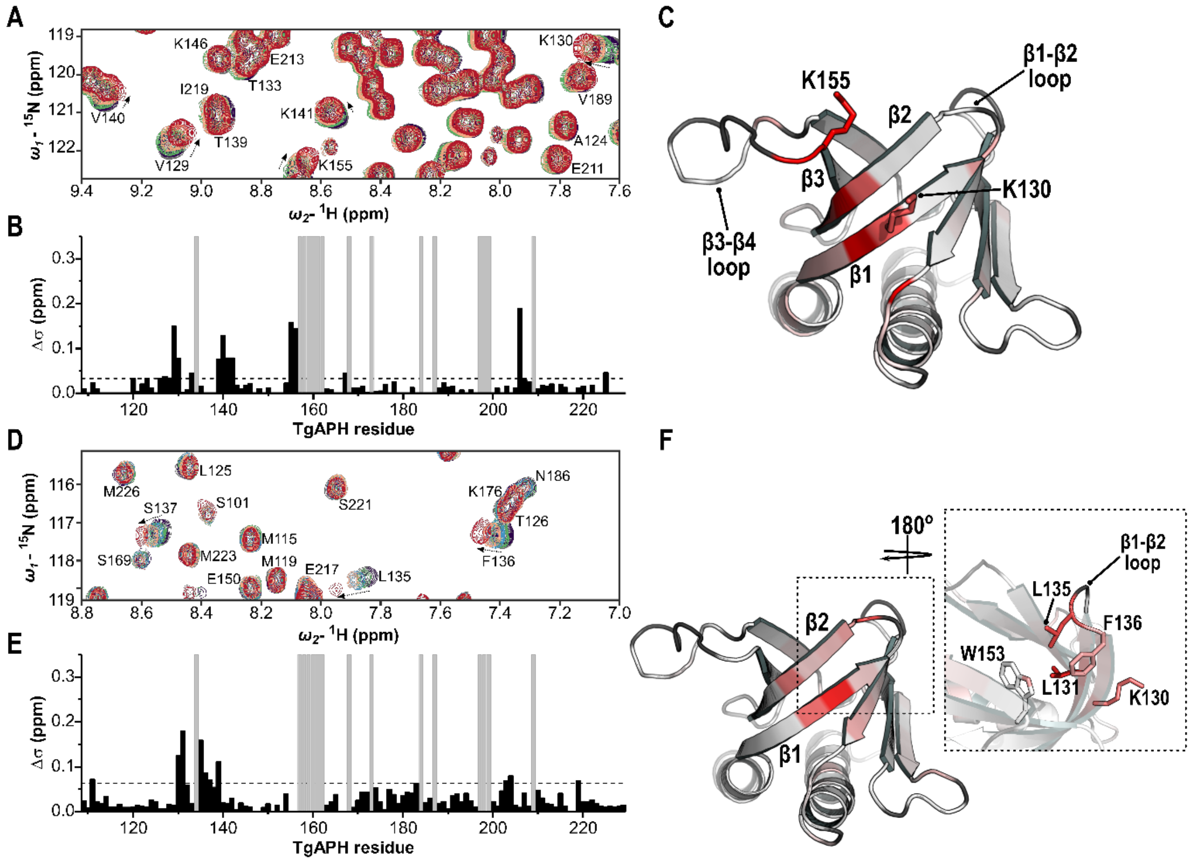

SI Figure S3 **(Related to Figure 2). Mapping the TgAPH$_{99\text{-}229}$:PA interface.** A) Overlay of representative ^{15}N-labelled TgAPH$_{99\text{-}229}$ 2D ^{1}H-^{15}N HSQC spectra recorded upon titration with increasing molar ratios of short-chain PA. HSQC spectra are coloured according to the molar ratio between ^{15}N-labelled TgAPH$_{99\text{-}229}$ and short-chain PA; *grey* 1:0, *purple* 1:1, *green* 1:3, *orange* 1:7, *red* 1:15. B) Plot of CSPs observed in A) upon titration with 15-fold molar excess of short-chain PA, versus TgAPH$_{99\text{-}229}$ sequence number. Residues that could not be assigned are indicated by a grey bar. Prominent CSP's are categorised as greater than 2σ's from the mean noise (0.032ppm), which is represented by a dotted line. C) CSPs observed in A) mapped onto the structure of TgAPH$_{99\text{-}229}$, coloured in a 20 interval *red* spectrum. A more intense colouring indicates a greater CSP as each interval represents 0.5σ from the mean noise. Key residues clustered around the β1 strand and β3-β4 loop region are labelled, unassigned residues are coloured *dark grey*. D) Representative data showing overlay of ^{15}N-labelled TgAPH$_{99\text{-}229}$ 2D ^{1}H-^{15}N HSQC spectra in the presence of increasingly PA enriched bicelles with the following DHPC:DMPC:POPA composition: *purple*, 75%:12.5%:12.5%; *green*, 75%:24%:1%; *blue*, 75%:18.75%:6.25%; *orange*, 75%:16.25%:8.75%; *red*, 75%:12.5%:12.5%. E) Plot of CSPs observed in D) upon titration with DHPC:POPC:POPA (75%:12.5%:12.5%) bicelles, versus PfAPH$_{106\text{-}235}$ sequence number, residues that could not be assigned are indicated by a grey bar. Prominent CSP's are categorised as greater than 2σ's from the mean noise (0.062ppm), which is represented by a dotted line. F) CSPs observed in D) mapped onto the structure of TgAPH$_{99\text{-}229}$, coloured as in C). Key residues clustered around the β1-β2 loop are labelled.

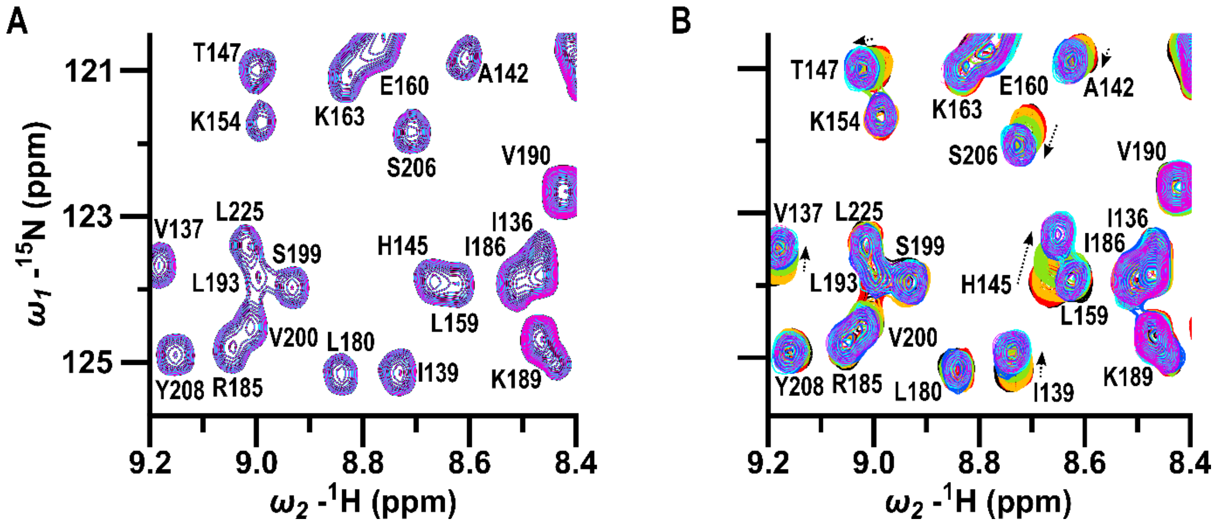

SI Figure S4 **(Related to Figure 2). APH does not bind PI($_{4,5}$)P$_2$ and is incapable of dual lipid recognition.** A) Overlay of representative ^{15}N- PfAPH$_{106-235}$ 2D ^{1}H-^{15}N HSQC spectra recorded upon titration with increasing molar ratios of phosphatidylinositol 4,5 bisphosphate (PI($_{4,5}$)P$_2$). HSQC spectra are coloured according to the molar ratio between ^{15}N-labelled PfA<u>PH</u> and PI($_{4,5}$)P$_2$; *black* 1:0, *cyan* 1:1, *purple* 1:2, *magenta* 1:4. HSQC spectra have decreasing line thickness to illustrate that peaks are overlaid. B) Overlay of representative ^{15}N-labelled PfAPH$_{106-235}$ 2D ^{1}H-^{15}N HSQC spectra recorded upon initial titration with increasing molar ratios of short-chain PA and then titration with increasing molar ratios of PI($_{4,5}$)P$_2$. HSQC spectra are coloured according to the molar ratio between ^{15}N-labelled PfAPH$_{106-235}$, short-chain PA and PI($_{4,5}$)P$_2$; *black* 1:0:0, *red* 1:1:0, *orange* 1:3:0, *lime* 1:7:0, *blue* 1:15:0, *cyan* 1:15:1, *purple* 1:15:2, *magenta* 1:15:8. Note that CSP's are only observed for peaks upon titration with short-chain PA.

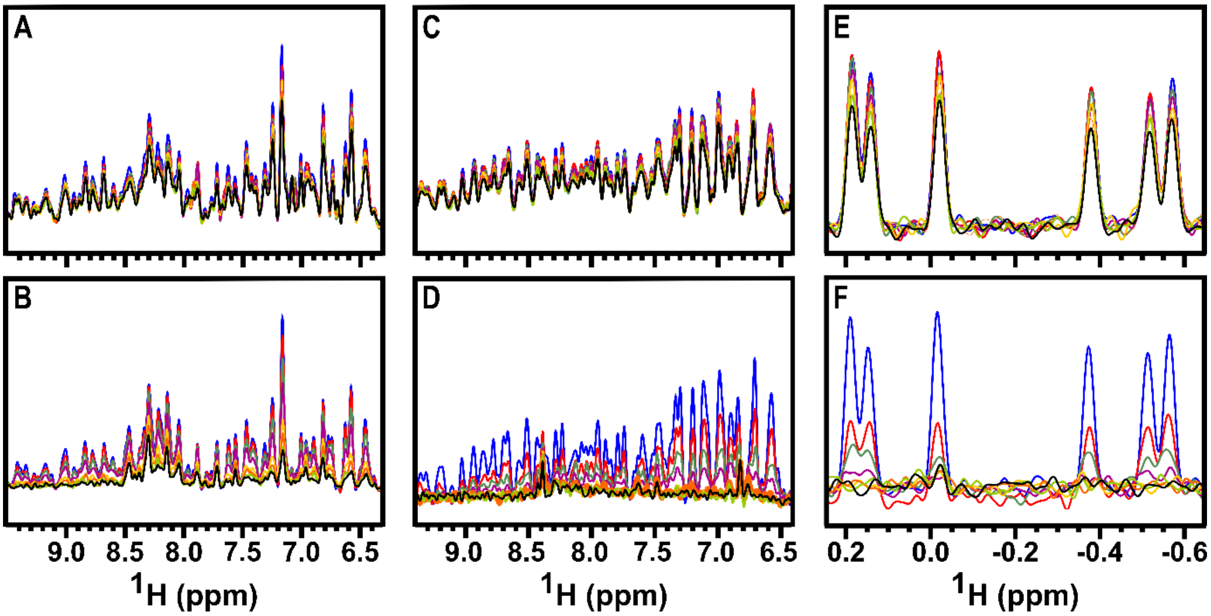

SI Figure S5 **(Related to Figure 3). APH titration with SUVs.** A) PfAPH$_{106\text{-}235}$ 1D ^{1}H NMR spectral region corresponding to the downfield-shifted amide region (9.5 to 6.3ppm) recorded upon titration with increasing concentration of SUVs composed of A) POPC (100%) or B) POPC and POPA (50%:50%). TgAPH$_{99\text{-}229}$ 1D ^{1}H NMR spectral region corresponding to the downfield-shifted amide region (9.4 to 6.4ppm) recorded upon titration with increasing concentration of SUVs composed of C) POPC (100%) or D) POPC and POPA (50%:50%). E) and F) identical to C) and D) but for TgAPH$_{99\text{-}229}$ 1D ^{1}H NMR spectral region corresponding to the upfield-shifted methyl region (0.25 to -0.65ppm). APH:SUVs (total lipid) molar ratio: *blue*, free APH in solution; *red* 1:2; *green* 1:4; *purple* 1:7; *yellow* 1:15; *orange* 1:20; *lime* 1:25; *black* 1:30.

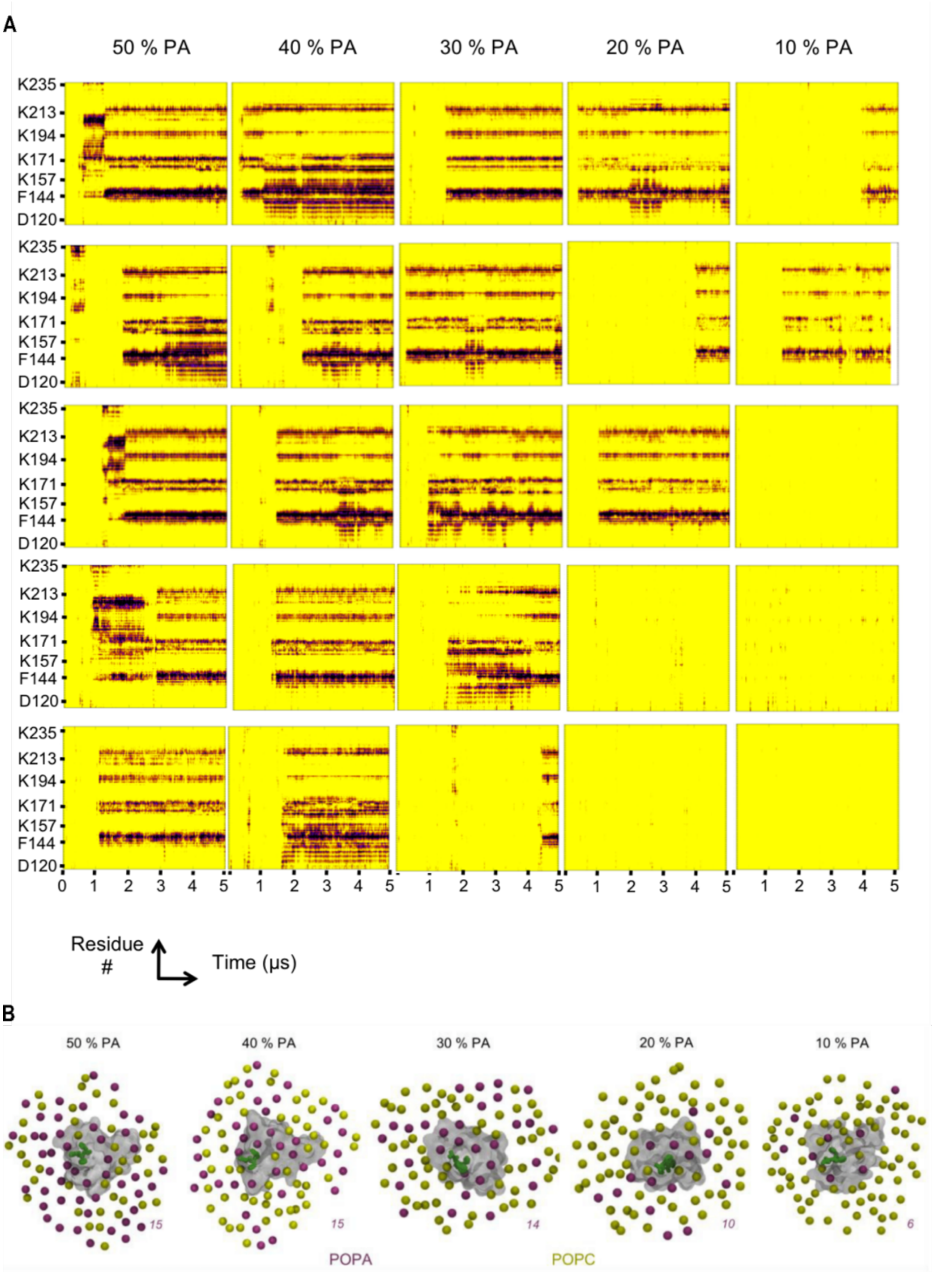

SI Figure S6 **(Related to Figure 6). Close contact plots between PfAPH$_{106\text{-}235}$ residues and POPA lipid headgroups, and clustering of POPA around the PfAPH$_{106\text{-}235}$ surface.** A) Residues are coloured purple when the distance from a POPA headgroup particle is below a cut-off of 1 nm, shown for 5 different simulations. The pattern of contacts between the protein and POPA is consistently observed at all 5 concentrations of POPA, indicating that PfAPH$_{106\text{-}235}$ predominantly adopts a single, stable orientation within the membrane. Upon finding the most favourable bound orientation the protein is not observed to depart the membrane for the remainder of the simulation time. B) Representative snapshots at each membrane composition (50- 10% PA) of the bound protein and lipid headgroup phosphate particles are shown. POPA headgroups are shown as purple spheres and POPC in yellow. The protein is shown as a grey surface with the buried hydrophobic residues I143-H145 shown as green ball and sticks. The POPA headgroups (purple) are observed to cluster together in the region of PfAPH$_{106\text{-}235}$ at all POPA concentrations. The maximum number of POPA within 1 nm of the protein surface for each PA composition is shown.

SI Table S1 **(Related to STAR Methods). Plasmids used in this study.**

Plasmid	Description	Source
For expression of full length APH and C-terminal PH domain		
pNIC28a-Bsa4_*PfAPH*$_{106\text{-}235}$	*6xHis-TEV-PfAPH*$_{106\text{-}235}$	This paper
pNIC28a-Bsa4_*TgAPH*$_{99\text{-}229}$	*6xHis-TEV-TgAPH*$_{99\text{-}229}$	This paper
pNIC28a-Bsa4_*TgAPH*$_{22\text{-}229}$	*6xHis-TEV-TgAPH*$_{22\text{-}229}$	This paper
For functional characterization of TgAPH in T. gondii		
iKD-GAC-DHFR	iKD-template	(Jacot et al., 2016)
pSAG1::CAS9-GFP-U6::sgUPRT	UPRT-Cas9 guide	(Shen et al., 2014)
pSAG1::CAS9-GFP-U6::sg*TgAPH*	*TgAPH*-Cas9 guide	This paper
pT8-N21-Ty-APH-BleO	Intermediate plasmid	(Bullen et al., 2016)
5'UPRT-pT8-MycGFPPfMyoAtail-Ty-3'UPRT	Intermediate plasmid	(Jacot et al., 2016)
pTub5-CAT	Intermediate plasmid	(Kim et al., 1993)
5'UPRT-CAT-pT8-N21-Ty-APH-3'UPRT	*TgAPH*-N21Ty	This paper
5'UPRT-CAT-pT8-N21-Ty-APH-K130A+K132A-3'UPRT	*TgAPH*-N21Ty-K130A+K132A	This paper
5'UPRT-CAT-pT8-N21-Ty-APH-L135A+F136A-3'UPRT	*TgAPH*-N21Ty-L135A+F136A	This paper
5'UPRT-CAT-pT8-N21-Ty-APH-K155A+K157A-3'UPRT	*TgAPH*-N21Ty-L155A+F157A	This paper
5'UPRT-CAT-pT8-N21-Ty-APH-E138A-3'UPRT	*TgAPH*-N21Ty-L138A	This paper
5'UPRT-CAT-pT8-N21-Ty-APH-Δ-linker-3'UPRT	*TgAPH*-N21Ty-Δ-linker	This paper
5'UPRT-CAT-pT8-N21-Ty-APH-Sc-linker-3'UPRT	*TgAPH*-N21Ty-Sc-linker	This paper

SI Table S2 **(Related to STAR Methods). Oligonucleotides used in this study.**

Oligonucleotide	Sequence (5'→3')
For expression of full length APH and C-terminal PH domain	
Fw_PfAPH	TACTTCCAATCCATGAAACTGAGCACCGAT
Rv_PfAPH	TATCCACCTTTACTGTCATTTCATGCTCATAATTTTGC
Fw_TgAPH	TACTTCCAATCCATGTCTGAACCTGACAACG

Rv_TgAPH	TATCCACCTTTACTGTTATTTCATCGACATGAACT
Fw_TgAPH_fl	TACTTCCAATCCATGCACATCAAAGCGAAAACC
Rv_TgAPH_fl	TATCCACCTTTACTGTCATTTCATCGACATGAACTTCAT
Site directed mutagenesis of APH PH domain	
PfAPH_K138A_K140A_Fw	TGCAACCGCGATTTTTCATGAAAC
PfAPH_K138A_K140A_Rv	ATTGCCACAATTTTGGTCAGGGTTTTG
PfAPH_K163A_K165A_Fw	CGCAAACGATAGCGATGGCAAA
PfAPH_K163A_K165A_Rv	CCTGCATACCATTCCAGCATTTTG
PfAPH_I143A_F144A_Fw	TAAAACCGCGGCTGCTCATGAAACCGTG
PfAPH_I143A_F144A_Rv	ATTTTCACAATTTTGGTCAG
PfAPH_H145A_Fw	CGCGATTTTTGCTGAAACCGTGAAAG
PfAPH_H145A_Rv	GTTTTAATTTTCACAATTTTGGTC
PfAPH_E146A_Fw	GATTTTTCATGCAACCGTGAAAG
PfAPH_E146A_Rv	GCGGTTTTAATTTTCACAATTTTG
TgAPH_K130A_K132A_Fw	CGCGACACACCTCTTTTCTGAG
PfAPH_K130A_K132A_Rv	AGCGCGACAACTTTAGTAAGAGCG
TgAPH_K155A_K157A_Fw	CAAGACACACGCCGCTTCTGAGACAGTGAAG
TgAPH_K155A_K157A_Rv	AGCTTGACAACTTTAGTAAG
TgAPH_L135A_F136A_Fw	CAAGACACACGCCGCTTCTGAGACAGTGAAG
TgAPH_L135A_F136A_Rv	AGCTTGACAACTTTAGTAAG
TgAPH_E138A_Fw	CTCTTTTCTGCGACAGTGAAGG
TgAPH_E138A_Rv	GTGTGTCTTGAGCTTGAC
Functional characterization of TgAPH in T. gondii	
CAT-Rev_2170	GCCCCGCCCTGCCACTCATCGC
M13-Rev_4749	AACAGCTATGACCATG
gRNA_4883	AACTTGACATCCCCATTTAC
TgAPH_6324 (iKD)	TCCCCACTGCGCATTATTTGCTTCCACTTCATGTTTGCGGATCCGGGG

TgAPH_6325 (iKD)	ACCTGTCAAAGCAGCTCATAGTGTTCCCCATTTTGATATCCCTAGGAATTCAC
TgAPH_6326 (APH CRISPR guide)	GCGTCGCTGAACCCGCAGTAGTTTTAGAGCTAGAAATAGC
TgAPH_6339Fw (K130A+K132A)	CGCGACACACCTCTTTTCTGAG
TgAPH_6529 Rv (K130A+K132A)	AGCGCGACAACTTTAGTAAGAGCG
TgAPH_6341 Fw (L135A+F136A)	CAAGACACACGCGGCGTCTGAGACAGTGAAGG
TgAPH_6342 Rv (L135A+F136A)	AGCTTGACAACTTTAGTAAG
TgAPH_6343 Fw (K155A+K157A)	TGCGAGTACCGCCGGCGCCCAGGAC
TgAPH_6344 Rv (K155A+K157A)	CCCGCGAACCACTGAACCTCTTCTCCATCTTTGCTG
TgAPH_6423 Fw (linker deletion)	TCTGAACCTGACAACGATGCGG
TgAPH_6424 Rv (linker deletion)	GGGAGCAGCGGCTCCAGG
UPRT_6611	CTCAAGTCTCAAAAGCAGATCCGC
UPRT_6610	ATCCCCTTCATTTTGCTTACGCAG
TgAPH _7327 Rv (E138A)	TCTTTTCTGCGACAGTGAAGG
TgAPH _7368 Rv (E138A)	AGAAAAGAGGTGTGTCTTGAGCTT
TgAPH _7399 Fw (Sc-linker)	CCCCCCGGGGATCTGCGCAGCAGCCAGATGGAAGCGAAAATGCGCTATGATGAAAAATCTGAACCTGACAACGATGCGG
TgAPH_7400 Rv (Sc-linker)	CCCCCCGGGTTCGCGCACGCTGCTGGTCGGGCGCATGCTTTCCAGGGTTTTGCTGCTGGGAGCAGCGGCTCCAGG

***SI Table S3* (Related to STAR Methods). 1D ^{1}H-NMR LUV titration experiments.**

Volume of 8mM LUV stock added to NMR sample (µl)	LUV concentration (µM)	APH:LUV ratio
0.0	0	1:0
6.9	100	1:2
7.0	200	1:4
10.6	350	1:7
28.7	750	1:15
18.8	1000	1:20
19.4	1250	1:25
20.0	1500	1:30
